# Genome-wide comparison of Asian and African rice reveals high recent activity of DNA transposons

**DOI:** 10.1186/s13100-015-0040-x

**Published:** 2015-04-28

**Authors:** Stefan Roffler, Thomas Wicker

**Affiliations:** Institute for Plant Biology, University of Zürich, Zollikerstrasse 107, CH-8008 Zürich, Switzerland

**Keywords:** DNA transposon activity, Rice, Proliferation mechanism

## Abstract

**Background:**

DNA (Class II) transposons are ubiquitous in plant genomes. However, unlike for (Class I) retrotransposons, only little is known about their proliferation mechanisms, activity, and impact on genomes. Asian and African rice (*Oryza sativa* and *O. glaberrima)* diverged approximately 600,000 years ago. Their fully sequenced genomes therefore provide an excellent opportunity to study polymorphisms introduced from recent transposon activity.

**Results:**

We manually analyzed 1,821 transposon related polymorphisms among which we identified 487 loci which clearly resulted from DNA transposon insertions and excisions. In total, we estimate about 4,000 (3.5% of all DNA transposons) to be polymorphic between the two species, indicating a high level of transposable element (TE) activity. The vast majority of the recently active elements are non-autonomous. Nevertheless, we identified multiple potentially functional autonomous elements. Furthermore, we quantified the impacts of insertions and excisions on the adjacent sequences. Transposon insertions were found to be generally precise, creating simple target site duplications. In contrast, excisions almost always go along with the deletion of flanking sequences and/or the insertion of foreign ‘filler’ segments. Some of the excision-triggered deletions ranged from hundreds to thousands of bp flanking the excision site. Furthermore, we found in some superfamilies unexpectedly low numbers of excisions. This suggests that some excisions might cause such large-scale rearrangements so that they cannot be detected anymore.

**Conclusions:**

We conclude that the activity of DNA transposons (particularly the excision process) is a major evolutionary force driving the generation of genetic diversity.

**Electronic supplementary material:**

The online version of this article (doi:10.1186/s13100-015-0040-x) contains supplementary material, which is available to authorized users.

## Background

Transposable elements (TEs) are found in practically all eukaryotes and are thought to have co-evolved with cellular life. Due to their virus-like lifestyle, TEs are considered ‘parasitic’ or ‘selfish’ DNA. However, recent studies revealed more detail about their role as potent genome shapers [1-4]. Most generally, TEs can be divided into two major classes such as: Class I (retrotransposons) and Class II (DNA transposons). Each class is further subdivided into several superfamilies [5]. For this study, we used the proposed classification system where each superfamily was assigned a 3-letter code [5] which will be given in parenthesis.

Retrotransposons use a mRNA intermediate that is reverse transcribed and integrated somewhere else in the genome. Therefore, each successful transposition produces an additional copy, which can lead to massive genome expansions [2,6]. In contrast, DNA transposons use a *cut and paste* mechanism to transpose and multiply. Because DNA transposons of the terminal inverted repeat (TIR) order [5] are the main focus of this study, we will describe their characteristics in more detail. In most TIR superfamilies, the pivotal transposase is flanked by TIRs and is transcribed and translated by the host machinery. *Mariner* (*DTT*) elements are, in copy numbers, the most abundant DNA transposons in rice and other grasses such as Sorghum [7] or Brachypodium [8] and usually encode a single transposase protein containing a catalytic DDD/E motif as do elements of the *hAT (DTA)* and *Mutator (DTM)* superfamilies. In contrast, *Harbinger (DTH)* and *CACTA (DTC)* elements also encode a second open-reading frame (ORF) of yet unknown function. Additionally, *CACTA* elements often contain complex arrays of subterminal repeats and large arrays of low complexity repeats which make them difficult to assemble and annotate [9].

Many transposons have lost their ability to transpose on their own. These non-autonomous elements usually lack protein-coding domains and transpose by recruiting enzymes of active, full size ‘mother’ elements. Such trans-acting systems have been described in both DNA [10] and retrotransposons [11]. Often, the non-autonomous elements, by far, outnumber their full-length counterparts and can represent a substantial amount of DNA in some genomes [8,12]. For example, in *Brachypodium*, 20,994 non-autonomous *Mariner* (*DTT*) elements were found whereas only 50 putative mother elements were identified [3]. Small non-autonomous DNA transposons are often referred to as ‘Miniature Inverted Transposable Elements’ (MITEs, [13,14]). Since we found non-autonomous elements of various sizes in multiple superfamilies, we prefer not to use the term MITE but rather refer to them simply as non-autonomous elements.

So far, several active DNA transposons have been found and documented in rice. The first described element was the non-autonomous, low copy element *mPing* of the *Harbinger (DTH)* superfamily [15-17]. One study identified *mPing* through mutability of a slender mutation of the glume which was caused by the insertion of *mPing* into the *slg* locus [15]. Kikuchi *et al*. identified *mPing* by a computational approach and presented a putative corresponding autonomous element which they named *Ping* [16]. Moreover, they showed experimentally that the transposition of both *mPing* and *Ping* preferentially occurs in cells derived from germ-line cells. Jiang *et al*. identified an additional, more distantly related autonomous element (*Pong*) which can activate *mPing in trans* [17]. Moreover, they could show experimentally that *mPing* preferably inserts in single-copy sequences. The *mPing*/*Pong* system has later been shown to transpose when introduced in heterologous systems such as in yeast [18] or Arabidopsis [19]. In 2005, Fujino *et al*. identified a non-autonomous element of the *hAT (DTA*) superfamily, *nDart*, that causes an *albino* phenotype and its putative autonomous mother element, *Dart*, which shared identical TIRs and similar subterminal sequences [20]. Finally, another member of the *hAT (DTA)* superfamily, *dTok*, was found to have inserted into the kinase domain of *FON1* during the molecular analysis of the *fon1/mp2* mutant [21]. Also here, they propose a putative autonomous element providing the necessary enzymes for the mobility of *dTok*. Interestingly, also in this study, transposon activity was found only in regenerative tissue.

Upon insertion, the host’s DNA is cut similar to a restriction enzyme, generating 3′ overhangs. After the transposable element (TE) has been inserted, these overhangs get complemented by the host’s repair system on both sides of the TE which leads to a duplication of the original target site. The length of this target site duplication (TSD) is an important diagnostic feature to classify DNA transposons, especially non-autonomous ones which do not encode any proteins (Table 1).

**Table 1:** **Target site characteristics of DNA transposon superfamilies**

**TE superfamily**	**Target site motif**	**Target site size**	**TIR consensus**
*Mariner (DTT)*	TA	2	CTCCCTC
*Harbinger DTH)*	TAA/TTA	3	GG(G/C)CC
*Mutator (DTM)*	Variable	9	GAG
*CACTA (DTC)*	Variable	3	CACT(A/G)
*hAT (DTA)*	Variable	8	CA

The current model of transposon excision proposes initial binding of the transposase to the TIR sequences followed by sequential cleavage of the two DNA strands. Thereon, dimerization of the paired-end complex brings the two strands in close proximity and links them by a clamp-loop protein [22]. Most likely, at least two subunits of the transposase (one binding to each TIR) are required for cleavage at the border of the element. When DNA transposons excise, they leave a double-strand break (DSB) with small 3′ overhangs which are derived from the TIRs of the element [22] (Additional file 1: Figure S1). Since DSBs are lethal for dividing cells, they need to be repaired by the host’s DSB repair systems. The applied repair pathways and therefore the footprint of the excision can vary substantially between species. There are two main groups of DNA repair pathways [23-25]. Which of the different pathways is applied depends on the cell-cycle phase and the nature of breakpoint ends. The simplest way of DSB repair is that the 3′ overhangs get denatured by exonucleases. This generates blunt ends which allow direct ligation of the two strands, called non-homologous end joining (NHEJ). These cases result in what is referred to as ‘perfect excision’ where only the TSD remains as a footprint [3] (Additional file 1: Figure S1A). The second major pathway uses short homologous sequences as templates to connect the two strands. These processes employ exonucleases to produce 5′ overhangs which resect until the newly exposed strands find a homologous region of a few bp between each other, allowing annealing of the overhangs. This is referred to as microhomology-mediated end joining (MMEJ) or single strand annealing (SSA). As a consequence, the sequence downstream of the homology will be lost resulting in a deletion (Additional file 1: Figure S1B). In some cases, if the homologous pattern that re-ligates the two strands corresponds exactly the complementary target site, this can lead to a restoration of the initial, ‘empty-site’ situation even before insertion of the TE (Additional file 1: Figure S1D). Such ‘precise’ excisions have been described to occur frequently when introducing the *mPing*/*Pong* system into Arabidopsis [19]. Thus, it is important to note that precise excisions are indistinguishable from insertions purely by means of comparative analysis. Alternatively, ectopic recombination can be initiated, which is referred to as synthesis-dependent strand annealing (*SDSA*). This can lead to the introduction of copies of foreign segments as ‘filler’ DNA (Additional file 1: Figure S1C). SDSA is also the mechanism underlying gene conversions [26]. In some cases, combinations of SSA and SDSA are utilized at the breakpoint leading to chimeric repair patterns [25]. While TE insertions or precise excisions are relatively easy to identify (*via* TSD), in some cases, it can be very difficult to precisely decipher excision footprints. Buchmann *et al*. [3] suggested that excisions of DNA transposons often cause extensive deletions which may also be combined with the introduction of foreign filler DNA.

In this work, we compared the genome sequences of Asian rice, *Oryza sativa ssp. japonica*, and African rice, *Oryza glaberrima*, whose genome sequence recently became available [27]. They diverged only about 600,000 years ago, providing an excellent opportunity to study recent TE activity and fixation. Moreover, it provided insight into the insertion and excision footprints, allowing inferring of qualitative and quantitative differences between TE superfamilies populating the two rice genomes. We aligned more than 63% of the two genomes and investigated 1,821 polymorphisms manually. Among these, we identified 487 loci with polymorphic DNA transposons that either inserted or excised since the divergence of the two species. We therefore estimate that the two rice genomes contain approximately 4,000 such polymorphisms. Moreover, we found differences in the excisions between different TE superfamilies. These seem to cause a multitude of rearrangements; some may be so dramatic that they cannot be detected at all anymore**.**

## Results

### TE families are unequally distributed within superfamilies

The assembled genome sizes (excluding Ns) are 372 Mbp for *O. sativa* (Version 5) [28] and 303 Mbp for *O. glaberrima* [27]. We were able to align approximately 63% of the two genomes (see below). We focused this study on DNA transposons of the TIR order (that is, elements are flanked by terminal inverted repeats and move with the help of a transposase enzyme). To obtain an overview of the abundance of DNA transposon families that had been active since the divergence of *O. sativa* and *O. glaberrima*, we used a database that was created based on an iterative search of insertion/excision polymorphic sequences in the alignments of the two genomes (see below and ‘Methods’). Thus, the results of our survey do not reflect the total content of DNA transposons in rice which, in fact, might be much higher [27]. We identified 64,645 Class II transposons of the TIR order in *O. sativa* and 54,280 in *O. glaberrima*, occupying approximately 20.4 Mbp and 12.6 Mbp of the two genomes, respectively (Additional file 2: Table S1). The average sizes of 316 bp and 230 bp reflect the strong outnumbering of autonomous by non-autonomous elements. A closer investigation of the substantial number of unclear sites (Ns) indicated that many sequence gaps in the *O. glaberrima* assembly are caused by Class II transposons (see ‘Methods’). We estimate that at least 5,100 sequence gaps actually correspond to Class II TIR elements, resulting in an estimated total of approximately 59,500 elements (approximately 16 Mbp) in *O. glaberrima*. Therefore, the overall DNA transposon content in *O. glaberrima* is probably slightly lower than in *O. sativa.*

**Figure 1:**
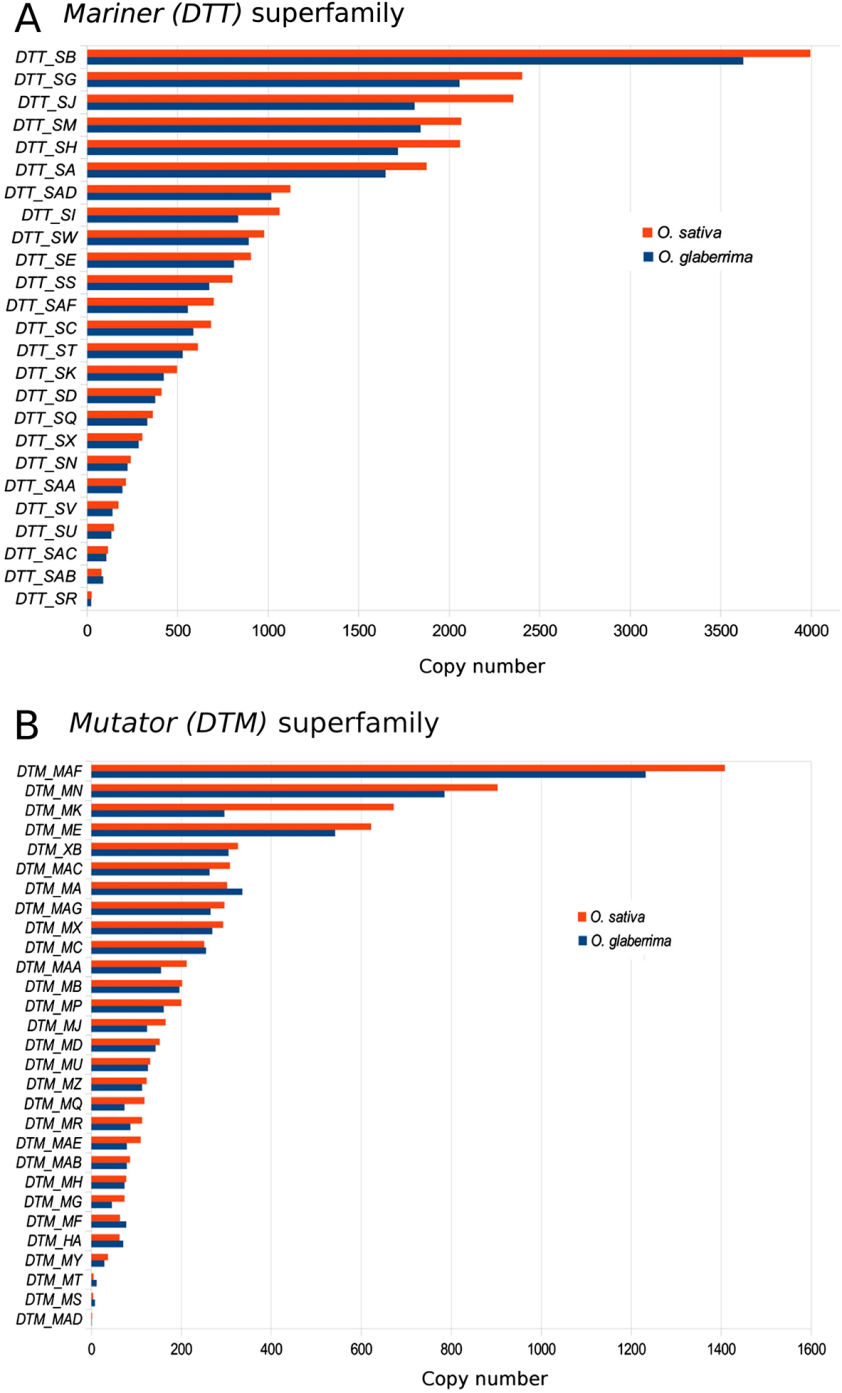
The abundance of *Mariner* (*DTT*) and *Mutator (DTM)* families in *O. sativa* and *O. glaberrima.* **(A)** Overview of *Mariner (DTT)* abundance. Copy numbers of individual families show large differences within the *Mariner (DTT)* superfamily. For example, in *O. sativa*, the most successful *DTT_SB* is represented 4′702 times while we only identified 25 copies of the *DTT_SR* family. **(B)** Overview of *Mutator (DTM)* superfamily. Despite an overall similar distribution, we found one exception for the *Mutator (DTM)* superfamily *DTM_MA*, where we found slightly more elements in the *O. glaberrima* genome (302 copies in *O. sativa* and 336 copies in *O. glaberrima*)*.*

In both species, the highest copy numbers were found for *Mariner* (*DTT*) elements, followed by elements of the *Harbinger (DTH)* superfamily. CACTA elements are on average larger than the other superfamilies (938 bp in *O. sativa* and 600 bp in *O. glaberrima*) and thus they occupy the most space. These findings are also consistent on the family level (that is, among elements that can be aligned at the DNA level [5]). We found strong over representation of a few families that dominate each of the superfamilies (Figure 1 and Additional file 2: Table S1). With the exception of the *DTA_Coraline* family (which was only found in *O. sativa*), all TE families are represented at similar numbers in both genomes (Additional file 2: Table S1).

### Identification of transposon polymorphisms

We were able to align 63.3% (235.6 Mbp) of the *O. sativa* and *O. glaberrima* genomes with the Smith-Waterman algorithm in sliding windows of 12 kb (see ‘Methods’) and examined the presence/absence of polymorphisms larger than 50 bp. We identified 23,709 polymorphisms in the *O. sativa* genome of which 7,542 showed homology to DNA transposons. In *O. glaberrima*, we found 22,003 polymorphisms whereof 4,816 had homology to DNA transposons. Upon visual inspection, we noticed that many of the polymorphisms in *O. glaberrima* that showed homology to TEs also contained large stretches of Ns, indicating that TEs are often problematic to assemble completely. Thus, in an independent approach, we estimated how many of the ‘presence’ polymorphisms in *O. glaberrima* which are comprised mostly of Ns actually correspond to TE sequences (see ‘Methods’). We estimate that there are approximately 1,750 polymorphisms in *O. glaberrima* which can be attributed to DNA transposons but which are not identifiable because the sequence assembly is incomplete at these sites. Thus we extrapolate that there are approximately 6,500 presence polymorphisms in *O. glaberrima*, slightly fewer than the 7,542 presence polymorphisms in *O. sativa* (see ‘Methods’).

Here, it should also be noted that sequence alignments of large genomic regions often contain misalignments caused for example by the presence on non-homologous segments or by sequence gaps in one of the species. Automated examination of sequence alignments can therefore yield very noisy data. Thus, we decided to manually analyze a subset of the identified polymorphisms. In total, we manually analyzed 1,821 cases which showed homology to TEs, 844 from *O. sativa* and 977 from *O. glaberrima*, representing approximately 15% of all TE-related polymorphisms. Most of them turned out not to be directly associated with TE activity because many represent internal or partial deletions within the elements, which means that the missing sequence obviously was not caused by an insertion or excision of the respective transposon but by a mechanism unrelated to its activity (for example, template slippage, Additional file 3: Figure S2).

In *O. sativa*, we found 238 and in *O. glaberrima* 249 TE polymorphisms that were most likely caused by DNA transposon activity. A complete overview of all active transposons is provided in Additional file 4: Table S2. Thus, 28% and 25% of all presence/absence polymorphisms examined represent likely transposition events. For *O. sativa*, we therefore extrapolate that about 2,100 of the TE-related polymorphisms are actually caused by TE activity. The value for *O. glaberrima* is probably similar. Considering the estimated, unknown part of approximately 450 additional TE-related polymorphisms, we expect a slightly lower overall activity of 1,650 transposition events in *O. glaberrima*.

In *O. sativa*, the most abundant were *Harbinger (DTH)* and *Mariner (DTT)* elements with 95 and 90 transpositions, respectively. Moreover, we identified 33 *Mutator (DTM)* and 15 *CACTA (DTC)* elements to have transposed recently. Finally, we found five elements of the *hAT (DTA)* superfamily. Also in *O. glaberrima*, *Harbinger* (*DTH*) and *Mariner* (*DTT*) were the most prominent superfamilies with 110 and 102 transpositions, respectively. Additionally, we identified 32 *Mutator* (*DTM)*, four *hAT* (*DTA*), and a single *CACTA* (*DTC*) transposition (Table 2)*.*

**Table 2:** **Overview of recently active DNA transposons in** ***O. sativa*** **and** ***O. glaberrima***

**TE superfamily**	***O. sativa***	***O. glaberrima***
*Mariner (DTT)*	90	102
*Harbinger (DTH)*	95	110
*Mutator (DTM)*	33	32
*CACTA (DTC)*	15	1
*hAT (DTA)*	5	4

### Distinguishing insertions and excisions

**Figure 2:**
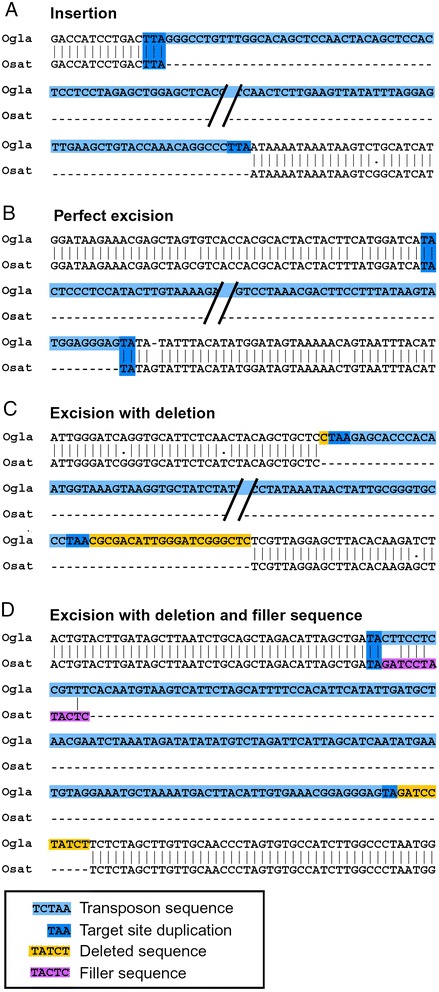
Examples of DNA transposon polymorphisms in *O. sativa (Osat)* and *O. glaberrima (Ogla)*. The alignments show the polymorphic TE plus some of the genomic flanking sequences. Diagnostic sequence motifs are *highlighted* with colors. **(A)** Insertion. **(B)** Perfect excision. **(C)** Excision with deletion. **(D)** Excision with deletion and filler sequence.

We defined TE insertions in the classic way as follows: one species contains the TE flanked by the two direct repeats created upon insertion (the TSD) while in the other species, the TE is absent and only one copy of the TSD is present (example in Figure 2A). Of the total 487 TE polymorphisms we identified in *O. sativa* and *O. glaberrima*, we classified 393 as insertions (192 in *O. sativa* and 201 in *O. glaberrima*). It is important to note that a precise excision (that is, one that removes the TE plus one target site) cannot be distinguished from an insertion with these criteria.

Excisions are much more complex to identify and show various patterns of DSB repair. The simplest case, a perfect excision, was defined as an event where the TE excises and exactly leaves the two copies of the TSD as a footprint [3]. Of a total of 94 putative excision events, we identified only eight perfect excisions (example in Figure 2B). In all other cases, the excision went along with deletions of flanking sequences or the insertion of filler sequences, or both. In 43 excisions, we found that sequences flanking the element were deleted. Excluding one extreme case (see below), on average approximately 18 bp of flanking sequences were deleted per excision event (example in Figure 2C). On the other hand, in 58 cases, excision also went along with the introduction of foreign DNA segments. On average these fillers had a size of 13 bp, ranging in size from 1 to 123 bp (example in Figure 2D). Nine cases showed both deletions and introduced filler segments. The cumulative length of all deleted sequences is 926 bp while the combined length of all filler segments is 880 bp.

**Figure 3:**
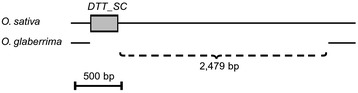
An example of a transposon excision that caused a large deletion in its flanking region. The transposon *DTT_SC* is indicated by a *gray box. Solid lines* represent the genomic sequences of *O. sativa* and *O. glaberrima*. The excision is precise at the left border of the *DTT_SC* element while a 2,479-bp segment was deleted at its right border.

The most extreme case was a putative excision of a *Mariner* element of the *DTT_SC* family. Its excision went along with the deletion of a 2,479 bp fragment on one side of the element (Figure 3). We are confident that this deletion was indeed the result of the excision because the left border of the excised fragment coincides precisely with the left end of the *DTT_SC* element (Figure 3). It is highly unlikely (however, not impossible) that a random deletion would have its one breakpoint exactly at the terminus of the TE. If this case is included in the overall calculation, a total of 3,405 bp were deleted in the 94 excision events.

### The ratio of insertions and excisions indicates differences in transposition between superfamilies

Inferring recent activity of DNA transposons from the numbers of excisions and insertions is not trivial. Intuitively, one would assume that the ratio of insertions and excisions is 1:1, because each excising element would simply insert somewhere else in the genome. The current hypothesis is that DNA transposons can excise during DNA replication and transpose in front of the replication fork to create an additional copy. This results in two different gametes, one with one copy and one with two copies. If the number of transposition events is large, overall equal numbers of loci derived from the two gamete types should be passed to offspring. Thus, if all the observed insertion/excision ratios in a cross-species comparison such as the one presented is considered, the ratio is actually expected to be 2:1 (Additional file 5: Figure S3). However, it is also possible that transposition happens at other points during the cell cycle, which would not lead to a replication of the respective TE. Considering this, one would therefore expect a ratio somewhere between 1:1 and 2:1 but not higher than 2:1. This 2:1 ratio is only expected if a given transposon family is active at similar levels in both species being compared. Deviation from the 2:1 ratio in the inter-species comparison would therefore indicate different levels of activity of that transposon family in the two species (Additional file 6: Figure S4). For example, a ratio much lower than 2:1 in *O. sativa* and much higher than 2:1 in *O. glaberrima* could indicate that a given transposon family was more active in *O. glaberrima* (Additional file 6: Figure S4).

When comparing the insertion/excision ratio for the different superfamilies, we observed almost the expected 2:1 ratio for the *Mariner (DTT)* superfamily. In both datasets, we found ratios which are not significantly different from 2:1 (2.6:1 in the *O. glaberrima* dataset and 2.8:1 in the *O. sativa* dataset), indicating that the proposed proliferation mechanism is sufficient to explain the observations on *Mariner* elements. It also indicates that precise excisions (removal of the TE plus one target site) are rare in the *Mariner* superfamily. Interestingly, for the *Harbinger* (*DTH*) superfamily, the ratio differs from what we expected. The ratio in the *O. glaberrima* dataset was 8.2:1 (Fisher’s exact test, *P* = 0.0001), and in the *O. sativa* dataset, we found 4.4:1 (*P* = 0.013). These differing ratios between *O. glaberrima* and *O. sativa* could indicate a higher level of *Harbinger* activity in *O. glaberrima*. Finally, we found a significant difference for the *Mutator* superfamily in the *O. glaberrima* dataset where we observed a ratio of 8.7:1 with 26 insertions and only three excisions (*P* = 0.03). The ratio in the *O. sativa* dataset (4.3:1), where we found 30 insertions and seven excisions, did not reach significance level (*P* = 0.14) (Table 3). It is not easy to explain why *Harbingers (DTH)* and *Mutator (DTM)* elements deviate so strongly from the expected 2:1 ratio in both species (see ‘Discussion’).

**Table 3:** **Overview of TE insertions and excisions by superfamily and species**

**TE superfamily**	**Species**	**Insertions**	**Excisions**	**Ratio**	***P*** **value**
***DTT***	*O. sativa*	59	21	2.81	0.19
***DTH***	*O. sativa*	84	19	4.42	0.01*
***DTM***	*O. sativa*	30	7	4.29	0.14
***DTC***	*O. sativa*	14	1	14	-
***DTA***	*O. sativa*	5	0	-	-
***DTT***	*O. glaberrima*	81	31	2.61	0.38
***DTH***	*O. glaberrima*	90	11	8.18	0.00008*
***DTM***	*O. glaberrima*	26	3	8.67	0.028*
***DTC***	*O. glaberrima*	0	1	-	-
***DTA***	*O. glaberrima*	4	0	-	-

*Significantly different from expected 2:1 ratio.

### High abundance does not necessarily correlate with strong activity

**Figure 4:**
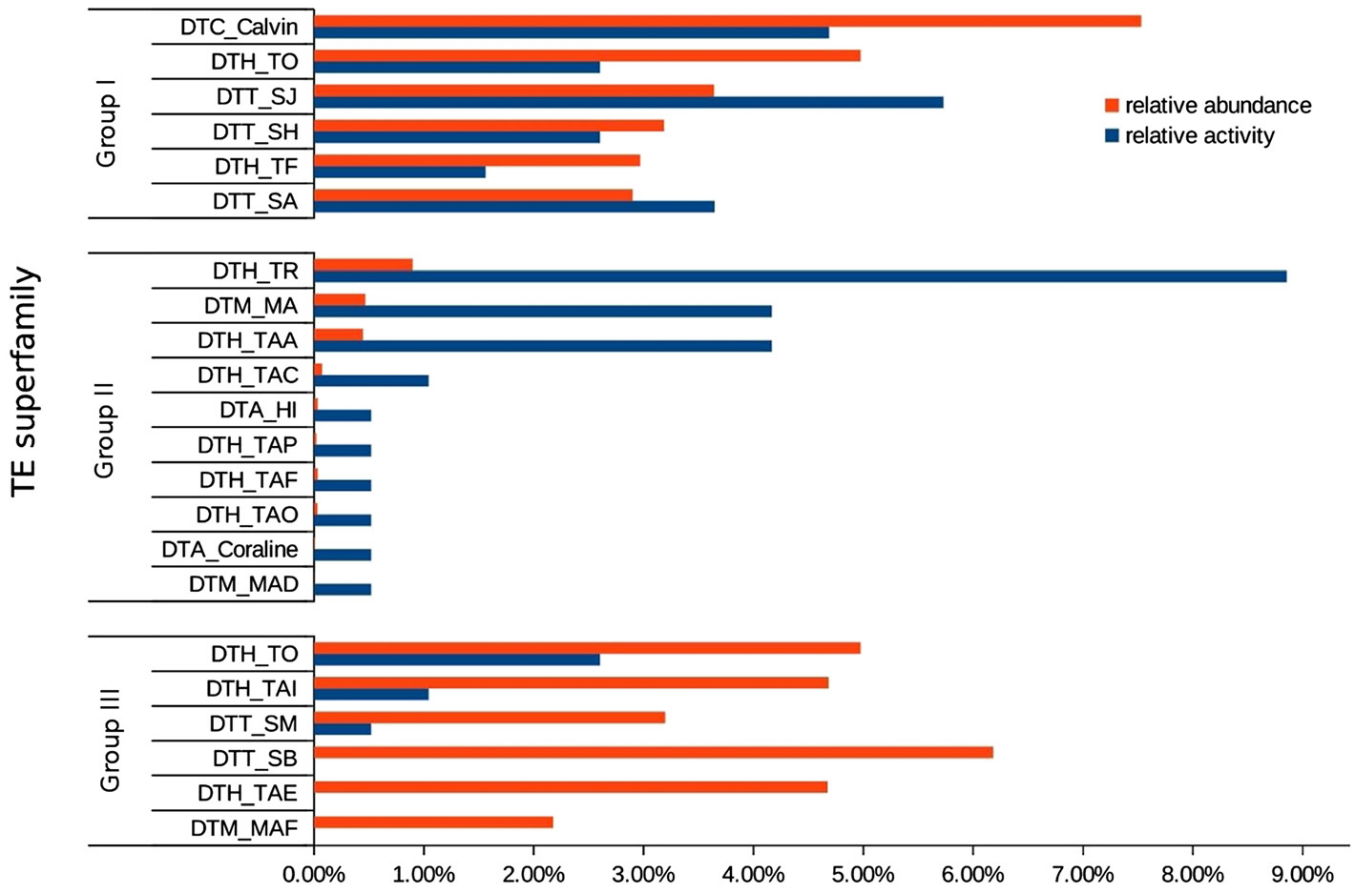
The relative activity and relative abundance of the TE families in *O. sativa.* We compared the relative activity with the relative abundance of all TE families in *O. sativa.* Group I consists of families with high activity and high abundance. The *CACTA* family *DTC_Calvin*, which is the overall most abundant family, also shows remarkable activity. Group II contains elements with high activity but low copy numbers. We found that *Mutator (DTM)* and *hAT (DTA)* families are relatively active despite their poor abundance. Finally, Group III consists of families with high abundance but relatively little activity. This class is dominated by families of the *Harbinger (DTH)* and *Mariner (DTT)* superfamilies. The *Harbinger* family *DTH_TO* seems to be still relatively active despite its high abundance, whereas the most abundant *Mariner* and *Mutator* families *DTT_SB* and *DTM_MAF*, respectively, show no activity at all.

To estimate activities of individual TE families, we had to consider that an additional sequence in *O. sativa* could mean that the TE inserted in *O. sativa* or excised in *O. glaberrima* and *vice versa*. Therefore, we had to combine data from both datasets. We defined the relative activity as the number of copies that moved in relation to the total copies in a particular TE family. As relative abundance we defined the total copy number of the respective family divided by the total number of DNA transposons of the investigated genome. We divided the families into three categories (Figure 4). In the first group, we grouped TE families with high overall copy numbers and also high numbers of insertion polymorphisms. Members of the *Mariner (DTT)* and *Harbinger (DTH)* superfamilies are most prominent in this category. Moreover, the most abundant *CACTA* family, *DTC_Calvin* (4,868 copies), turned out to be also very active with nine identified insertions and one excision.

The families in the second group show a high number of insertions relative to their abundance. Most noticeably, for the *DTH_TR* family, of which we found only 581 copies in the whole *O. sativa* genome, we identified 17 insertions and one excision, more than for any other family overall. Other highly active families in this class are the *Harbinger* family *DTH_TAA* and the *Mutator* family *DTM_MA* which both inserted eight times and excised once while we found 288 and 302 copies, respectively. Furthermore, we found several other *Harbinger (DTH)* and two *hAT (DTA)* families with less than 50 copies and one insertion. The most extreme case here is the *Mutator* family *DTM_MAD* where we only found two copies in the whole genome, one of them inserted recently.

The third group contains families with high abundance but with only little or even no activity. Here, we find the most numerous families, again of the *Harbinger (DTH)* and *Mariner (DTT)* superfamilies, where we found several families with more than 3,000 copies but only five or less polymorphisms. For the most numerous, *Mariner* family *DTT_SB* (3,995 copies), and the most abundant, *Mutator* family *DTM_MAF* (1,408 copies), we did not find any polymorphic elements at all (Additional file 2: Table S1 and Additional file 4: Table S2).

### Most potentially active and autonomous elements are of the *Mutator* superfamily

As mentioned above, the majority the elements that transposed since the divergence of the two rice species are non-autonomous elements which do not code for any proteins. We found a total of 17 elements which contain at least parts of transposase ORFs and have moved since species divergence. Interestingly, twelve of them belong to the *Mutator (DTM)* superfamily, which had, overall, relatively few active elements (see above). We found eight families where all polymorphic copies contain at least parts of coding sequences (CDS) for transposases (*DTM_MAF*, *DTM_MS*, *DTM_MAG*, *DTA_Coraline*, *DTA_HL*, *DTH_TAG*, *DTH_TAH*, and *DTH_Blip*). For the *Mutator* family *DTM_MU*, we found one CDS-containing element and one non-autonomous deletion derivative. However, besides the *DTA_Coraline* insertion, all the transposase ORFs of the above families have either stop codons or frameshifts, suggesting that they are not functional autonomous elements.

**Figure 5:**
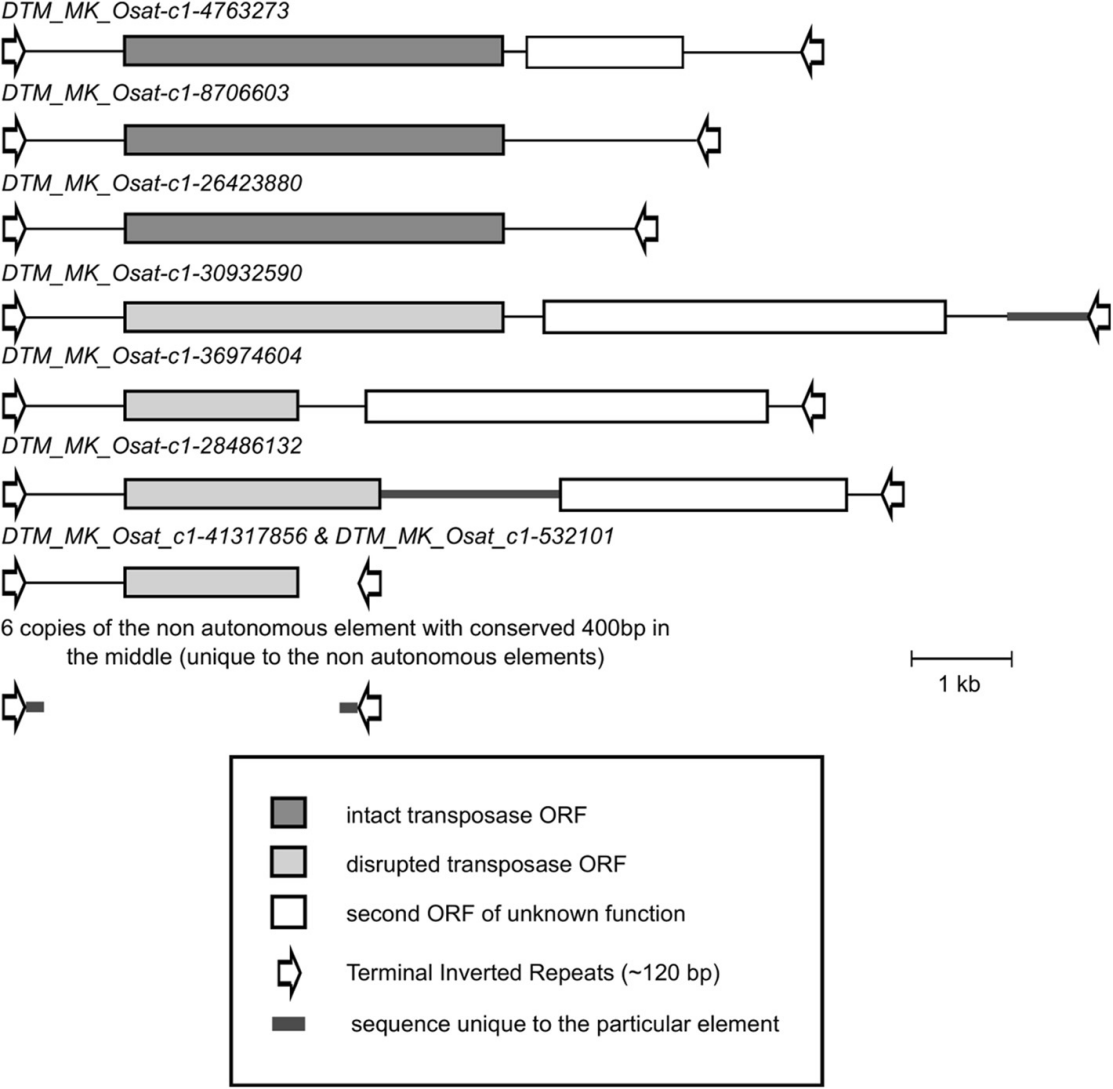
The schematic representation of the copies of the *Mutator* family *DTM_MK* which were polymorphic in *O. sativa* and *O. glaberrima.* The family includes three copies which contain intact transposase ORFs (top three copies). One of these putative mother elements additionally carries a fragment of a second ORF which was also found in other derivatives. Presumed non-autonomous copies have partially deleted or disrupted reading frames containing stop codons or frameshifts in the transposase ORF. Additionally, we found six copies of non-autonomous elements which consist only of TIRs plus an internal sequence that has no homology to that of larger elements (bottom). The fact that all six are very similar to each other indicates that they originate from the same deletion event and are multiplied later.

The most interesting *Mutator* family is *DTM_MK* which contains 14 elements that have moved since species divergence (Figure 5). We found a total of nine insertions and one excision of *DTM_MK* elements in *O. sativa* and four excisions but no insertion in *O. glaberrima*, indicating that they had been active in both species. Here, we found three elements with apparently intact transposase ORFs which we consider potentially active mother elements. The largest among these (6,721 bp) contains an intact transposase ORF and an additional ORF that encodes a ‘TE-associated’ protein. Interestingly, we found large parts of the same second ORF in three other putative full-length elements that all have disrupted transposase ORFs. Furthermore, two of the disrupted elements acquired an additional sequence which has no homology in any of the other family members (Figure 5). Intriguingly, we also found a sub-population of six non-autonomous elements that had moved. These elements are very similar to each other in size (604 bp to 684 bp) and share the TIRs of around 120 bp with the other larger elements. The approximate 400 bps between their TIRs is not homologous to any of the larger elements but is highly similar in the six small copies. This indicates that these six copies originated from a single deletion event and multiplied after (Figure 5).

### TE activity mainly influences regions close to genes but not coding sequence

We investigated if and to what extend TE activity affects genes by using the coding sequence provided by the Rice Genome Annotation Project [29]. We included all TE polymorphisms (insertions and excisions) found in exons as well as those in introns, 1,000 bp upstream and 500 bp downstream of the coding sequence.

Of the 487 investigated TE polymorphisms, 160 matched our criteria. We found 74 insertions or excisions in upstream regions and 24 downstream of genes. Moreover, 61 polymorphisms were identified in introns. Interestingly, only one, a *Mariner* element of the *DTT_SG* family, actually disrupted a gene in *O. sativa*. The element inserted into the second exon of a glutathione S-transferase homolog (LOC_Os01g72120), a protein assumed to be involved in detoxification. We furthermore performed a gene ontology analysis. This revealed that genes involved in nucleoside metabolic and biosynthetic processes, protein dephosphorylation and SRP-dependent proteins targeting the membrane, are affected disproportionately high (*P* < 0.01/not shown).

## Discussion

In this study, we conducted a genome-wide analysis of the activity of DNA transposons in the two closely related rice species *O. sativa* and *O. glaberrima*. Numerous studies have described the activity of (Class I) retrotransposons in plants [2,6,30,31], but only very few have focused on DNA (Class II) transposons. To our knowledge, this is the first study that characterizes DNA transposons and assesses their activity at a genome-wide scale. Here, the recently released sequence of the *O. glaberrima* genome [27] provided a unique opportunity for comparative analysis because it is phylogenetically close enough to *O. sativa* to allow reliable sequence alignments of large parts of the genome and yet distant enough to have accumulated numerous TE polymorphisms. Both the *O. sativa* and the *O. glaberrima* genome were sequenced with Sanger technology and assembled independently. This has the important advantage over simple re-sequencing and subsequent mapping onto a reference so that large insertions and deletions in both species can be easily identified and characterized in much detail. A very important part of our work was that we manually inspected over 1,800 TE related polymorphisms in the two species because transposon excisions, especially, can produce complex sequence patterns which are extremely difficult to characterize on an automated basis. Furthermore, it was important to distinguish actual insertions and excisions from random deletions that by chance affected parts of the TEs. The result of this study is a chromosome-scale catalog of TEs that were recently active in rice as well as information on how transposon insertions and excisions affect the genome. Our data allow conclusions and hypotheses on transposon activity. These will be discussed below.

### Estimates of frequencies of transposition events in *O. sativa* and *O. glaberrima*

It has been known for several years now that grass genomes contain tens of thousands of DNA transposons [7,12]. However, it was not clear how often these elements actually move. *Wessler et al.* [10] suggested that some families may be active in bursts, creating thousands of copies within only a few generations before they are silenced by the host. The data from our study now allow some conclusions on the actual level of transposition activity such that:. *O. sativa* and *O. glaberrima* were estimated to have diverged approximately 600,000 years ago [27]. Overall, our data indicate that DNA transposons were active at similar levels in both rice species since their divergence. Based on the manual analysis of insertions, we estimated that *O. sativa* contains roughly 2,000 polymorphic elements and *O. glaberrima* approximately 1,600. Assuming that most of these polymorphic transposons are actually fixed in the two species, we can estimate that since species divergence, a DNA transposon polymorphism (insertion or excision) became fixed approximately every 250 years to 300 years in both *O. sativa* and *O. glaberrima*. In other words, about 2.5% to 3.5% of the DNA transposons in the two species have moved within the last 600,000 years.

For the following calculations, we assume that all identified transposition events were selectively neutral as deleterious transpositions would have been selected against. However, fixed polymorphisms only represent a small part of actual TE activity. A measure for actual transposon activity can be defined analogous to a mutation rate (m) as the number of transposition per generation per individual. The total number of transposition events per generation would therefore be the effective population size N(*e*) times the mutation rate (that is, N(*e*)*m*). According to Kimura [32], fixation rates are inversely proportional to population sizes. Thus, if all transposition events are neutral, the probability of fixation of an event is 1/N(*e*). The rate of fixation is therefore N(*e*)*m* × 1/N(*e*) = *m*. Thus, population size is irrelevant, and the fixation rate is equal to *m* [32]. In the case of *O. sativa*, fixation rate would therefore be the number of identified transposition events (assuming all of them are fixed) divided by the number of generations since divergence from *O. glaberrima* (2,300/600,000 = 0.004). This would mean that in each generation, 1 out of 250 individuals contains a transposition event.

### Most polymorphic DNA transposons are non-autonomous, except in two superfamilies

The vast majority of the polymorphic transposons were small non-autonomous elements (MITEs) of the *Mariner (DTT)* and *Harbinger (DTH)* superfamilies. Interestingly, we did not find any polymorphic potentially autonomous elements for either of the two superfamilies. This could indicate that the required transposase genes may still be expressed, but the mother elements themselves have lost the ability to move. It was previously reported that non-autonomous *Mariner* and *Harbinger* elements could also be cross-activated by even distantly related mother elements and even in heterologous systems when non-autonomous rice elements are introduced into yeast and Arabidopsis [10,18,19].

We found polymorphic putative full-size elements of twelve *Mutator*, three *Harbinger* and two *hAT* families. However, even among these large elements, most carried defective transposase ORFs which contained frameshifts or stop codons. The only exceptions were an insertion of the *hAT* element *DTA_Coraline* and several members of the *Mutator* family *DTM_MK*. Here, we found multiple copies that contain intact transposase ORFs. The *DTM_MK* family is particularly interesting because it illustrates how TEs can diverge into multiple sub-families. The *DTM_MK* family consists of multiple large elements that each contains a unique pattern of internal deletions of additionally acquired sequence fragments. Furthermore, it contains a sub-population of six small deletion derivatives that obviously originated from a single deletion event since they all have a very similar structure. These elements may represent the first steps in the evolution of a population of non-autonomous TEs.

### DNA transposon excisions have a large potential to shape the genome

Of particular interest to us was a broad assessment of what types of footprints DNA transposons produce. We found that TE excisions can produce very complex patterns. Previous studies already suggested that excisions may produce a variety of outcomes and that the perfect footprint (that is, the precisely duplicated target site) might actually be rare [3]. Furthermore, it was shown that excisions may lead to large deletions and/or insertions of copies of foreign DNA fragments when ‘filler’ DNA is inserted in the process of DSB repair [3,16,33]. Our data indeed show that perfect excisions which leave exactly two copies of the TSD are extremely rare, as only 8 out of 94 excisions showed this pattern. In all other cases, excisions lead to the deletion and/or introduction of foreign DNA fragments. Our large dataset allowed us to quantify that on average, 18 bp of the flanking region are deleted while 13 bp of the new sequence are introduced at the excision site. These numbers do not include the most extreme case wherein an excision apparently went along with the deletion of a 2.4 kb fragment. Furthermore, our dataset does not include possible cases where large segments on both sides of the element were deleted upon excision (such events would be indistinguishable from random deletions that by chance removed a large segment containing the TE). Also data from insertion/excision ratios of some superfamilies suggest that many excisions may have ‘catastrophic’ outcomes (see below). Thus, we conclude that excisions of DNA transposons are a major driving force in genome evolution as they can cause relatively large-scale rearrangements such as deletions and integrations of new sequences surrounding the excision site.

### Why do *Harbinger* and *Mutator* elements show more insertions than expected?

The current model of proliferation during DNA replication postulates that one would find a ratio of insertions to excisions that lies somewhere between 1:1 and 2:1 (see Additional file 5: Figure S3 and Additional file 6: Figure S4) when comparing two closely related genomes. Interestingly, in all DNA transposon superfamilies, we found insertion/excision ratios higher than 2:1 in both species. Only for the *Mariner (DTT)* superfamily did we find a ratio of insertions to excisions that was only slightly above 2:1 in both *O. sativa* and *O. glaberrima*. In contrast, the insertion/excision ratios of the *Harbinger (DTH)* and *Mutator (DTM)* superfamilies are clearly higher than 2:1 (that is, they show a much higher number of insertions than expected). The same is also probably true for *CACTA (DTC)* elements, but there, the sample size is smaller and the insertion/excision ratio does not significantly deviate from 2:1.

One explanation for the distorted ratio is that, for some reason, we can simply not see excisions in our sequence alignments. Buchmann *et al.* [3] suggested that some excisions go along with deletions of several kb of the flanking regions. Indeed, for example for *Harbinger* elements we identified the highest proportion of “unclear” events. These comprise large sequence gaps which we could not clearly classify as excisions because too much sequence was deleted or rearranged surrounding the element. Thus, our hypothesis is that *Harbinger* and *Mutator* (and possibly *CACTA*) elements frequently cause large rearrangements (mostly large deletions) upon excision, so that the orthologous regions of the two species cannot be aligned easily anymore. If such deletions are in the size range of 3 kb to 5 kb, it would undermine our initial mapping of homologous loci that was based on blast searches of 5 kb segments. Additionally, if the fitness of a gamete carrying the excision is reduced or even lethal, this would also contribute to raising the ratio above the 2:1. One possible reason for frequent large deletions could be the size of the elements, simply because excisions of large elements may be more difficult to repair. Indeed, *Mariner* elements are on average the smallest of all the elements studied, and there, we find an insertion/excision ratio to be the closest to 2:1. With increasing average size of elements, we also see an increasing insertions/excision ratio.

A second explanation why we find fewer excisions than expected is that the DSB is repaired by using the sister chromatid as a template *via* the SDSA mechanism [34] analogous to what happens during gene conversion. In this case, the excision would be undetectable because it was repaired perfectly with a copy of the sister chromatid that still contains the insertion. Such reversion of excision sites has been described in *Drosophila melanogaster* [35] and *Caenorhabditis elegans* [36]. However, it is not clear why this repair mechanism would preferably be used in certain superfamilies such as *Harbinger* and *Mutator*.

Finally, it is possible that many excisions are precise (that is, the TE and one target site is removed) and thus could not be distinguished from insertions. This could, for example, explain our findings of the high ratios of 4.4:1 for *O. sativa* and 8.2:1 for *O. glaberrima* in the *Harbinger* superfamily. However, previous studies produced conflicting results on the frequency of precise excisions. Yang *et al*. [19] described that 83% of approximately all 30 excisions were precise for the *Harbinger* element *mPing* when expressing it in *A. thaliana*. In contrast, Kikuchi *et al*., who worked with the same element in rice anther cultures, stated that only one case out of approximately 70 excision sites showed the footprint of a precise excision [16]. Thus, it is possible that the frequency of precise excisions depends on the conditions under which the transposition occurs. Additionally, the frequency of precise excisions could also differ between TE superfamilies. Indeed, for Mariner elements, we found a ratio close to 2:1, indicating that we were able to distinguish insertions and excisions well.

## Conclusions

We conclude that the activity of DNA transposons (particularly the excision process) is a major evolutionary force driving the generation of genetic diversity. Additionally, our data indicate that some DNA transposon excisions might cause such large-scale rearrangements so that they cannot be detected anymore. It is therefore likely that our study still under-estimates the impact of DNA transposon excisions on genome evolution. However, it will require further and more detailed studies of these transposable elements in multiple species to conclusively answer this question.

## Methods

### Genome-wide sequence alignments

The genome of *O. sativa* was split into fragments of 5 kb. Each of these fragments were then used in BLASTN searches against the *O. glaberrima* genome to identify the orthologous regions. As a primary filter criteria, we considered only fragments in the same orientation on the same chromosome with an identity of at least 96%. Then, 12 kb of sequence from both species (5 kb fragment + 7 kb adjacent 3′ sequence to create an overlap with the following fragment) were excised for pair-wise alignment. Here, we used the EMBOSS (emboss.sourceforge.net) program Water which implements the Smith-Waterman algorithm. We used a gap opening penalty of 30 and gap extension penalty of 0.1 to obtain alignments that preferably contain fewer but larger gaps.

Each of these pairs was scanned for alignment quality. We included all sequences that were embedded between at least 200 continuous bases that could be aligned with more than 90% perfect matches. The corresponding positions in the *O. sativa* genome were determined, and the overlapping individual alignments were re-assembled into one global alignment per chromosome. The consistency of the global alignment with the original assembly of *O. sativa* was tested extensively by manual comparisons of positions of randomly chosen sequences in and across the breakpoints of the overlaps. The global alignments were scanned for insertions or deletions (InDels) larger than 50 bp. InDels only separated by less than 4 bp were considered as one event. Additionally, InDels that bordered to sequence gaps (stretches of Ns) were discarded.

The remaining InDels were scanned for homologies to Class II TIR-order transposons from our in-house database that is derived from the TREP database (http://wheat.pw.usda.gov/ITMI/Repeats/) with the following BLASTN parameters: minimum alignment size of 50 bp and identity of at least 70%. The InDels that could not be associated to known TEs were used as a query for an iterative BLASTN search against the whole genome in which the InDel was found. Sequences with at least 15 copies and a minimum identity of 85% were considered putative TEs. The top 15 hits were extracted from the genome including a few hundred bp of flanking sequences. These were aligned with ClustalW to determine the precise borders of the element and to generate a consensus sequence. Consensus sequences were curated manually and added to the repeat database. Like this, we were able to expand the existing dataset for rice repeats at TREP from 59 sequences to 235 sequences. All scripts were written in PERL and are available upon request.

The data for this analysis were retrieved from Wang *et al*. [27] for *O. glaberrima* and the International Rice Genome Sequencing Project (IRGSP) for *O. sativa Nipponbare cultivar* [20] (plantbiology.msu.edu/pub/data/), respectively. We retrieved the annotation of the *O. sativa* genome from the Rice Genome Annotation Project [29] (Version 6/plantbiology.msu.edu). We removed all entries that included the word ‘transpos’ in the description line as well as putative genes (which also mostly correspond to TE sequences) and mapped the remaining genes on our version (version 5) of the genome using GMAP [37] (research-pub.gene.com/gmap/). For the gene ontology analysis, we used the online platform ‘Rice Oligonucleotide Array Database’ [38] (ricearray.org/analysis/) at default settings. We included the genes found to be affected by active TEs in exons, introns, 1,000 bp upstream, or 500 bp downstream of the CDS to check if they are often involved in certain biological processes disproportionately.

### Estimate of the number of sequence gaps caused by DNA transposons

To assess the different assembly qualities, we first counted all Ns in both genomes. With a total of 93,930 Ns, the *O. sativa* assembly contains a low number of sequence gaps. In contrast, in *O. glaberrima*, we identified 20,080 gaps consisting of more than 50 Ns (total N count, 12,768,901). To study the cause of these sequence gaps, we extracted 500 bp up and downstream of these regions and identified the orthologous position in *O. sativa.* We identified 7,301 cases (4,413,818 Ns), where both flanking sequences mapped within 10 kb from each other in the same orientation (blast hits with a minimum of 400 bp length and 95% identity). We then screened the segment in *O. sativa* that corresponds to the gap in *O. glaberrima* for TE sequence. Of these orthologous loci, 25.6% (1,871 cases) showed homology to TIR DNA transposons. From this number, we extrapolated that proximately 5,150 sequence gaps in the *O. glaberrima* genome correspond to TIR DNA transposon sequences.

In the alignment of the two genomes, we identified 1,745 insertions (that is, additional sequence) in *O. glaberrima* larger than 50 bp which consist of more than 80% Ns. Assuming that about 25% of these loci correspond to DNA transposons, we expect 447 additional DNA transposon-related polymorphisms in *O. glaberrima*.

### Additional data files

The following additional data are available with the online version of this paper. Additional file 1 is an illustration of the different DSB repair mechanisms. Additional file 2 is a table listing all annotated DNA transposons in the genomes of *O. sativa* and *O. glaberrima*. Additional file 3 is a figure explaining different mechanisms that lead to InDels. Additional file 4 is a table listing all described TE polymorphisms. Additional file 5 is a figure explaining the inheritance of insertion and excision patterns of DNA transposons. Additional file 6 is a figure explaining that the differences in the ratio of insertions and excisions is an indicator for differential TE activity between species.

## Additional files

Additional file 1: Figure S1. Overview of the different mechanisms of DSB repair following DNA transposon excision. A.) Non-homologous end joining (NHEJ) which leads to perfect excisions. B.) Single stranded annealing (SSA) which leads to a deletion of adjacent sequences. C.) Synthesis-dependent strand annealing (SDSA) which can lead to introduction of ‘filler’ DNA segments. D.) A special case of SSA which leads to precise excisions. Additional file 2: Table S1. Overview of TE abundance in *O. sativa* and *O. glaberrima*. Additional file 3: Figure S2. Summary of causes for insertions in rice species. Many of the identified insertions showed homology with DNA transposons but were not caused directly by their activity (for example, partial deletions of TEs). Therefore, we divided the remaining insertions into three classes based on their presumed molecular mechanism as follows: (i) repeat slippage, (ii) partial deletion, and (iii) unknown. Repeat slippage happens if DNA polymerase loses its template while synthesizing the new strand during replication and then re-adopts at a similar template close by. We found 149 insertions in *O. glaberrima* and 51 in *O. sativa* which represent differences in the number of tandem repeats between the two species. Template lengths ranged from simple dinucleotides to more than 20 bp. In two cases, entire TEs served as templates for slippage, deleting several kb between two elements. In these cases, unequal homologous cross over (similar to the mechanism that produces solo LTRs of retrotransposons) could be an alternative interpretation. Another 68 insertions in *O. glaberrima* and 94 in *O. sativa* resulted from partial deletions of TEs. These were deletions of apparently random segments within or close to TEs. Finally, 35 insertions in *O. glaberrima* and 66 in *O. sativa* could not be clearly classified. These InDels are often larger than the average InDel. These include cases where it was not possible to deduce the original, ancient state because, for example, multiple TEs were nested in these positions. Also included here are cases where a TE was found in the middle if a large insertion. These could potentially represent excisions which went along with deletions of large segments of the flanking sequence. Additional file 4: Table S2. Overview of all transpositions. Additional file 5: Figure S3. Inheritance of transposon insertion/excision patterns. For this model we assume that all transposition effects are selectively neutral. It is commonly accepted that a mechanism of multiplication is for DNA transposons to excise during DNA replication and to reinsert in front of the replication fork. This leads to one daughter strand with one copy of the element (A-type gamete) and one with two copies (B-type gamete). If a large number of transposons are active in many different loci in a species (this may be spread out over many generations), the offspring genome will be a mosaic of loci derived from A- and B-type gametes. When comparing that genome to that of a closely related species, loci resulting from A-type gametes will identify an excision and an insertion, while loci resulting from A-type gametes will only identify insertions. Thus the observed overall ratio of insertions to excisions from a given transposon family will be 2:1. Additional file 6: Figure S4. Detection of differences in transposon activity in different species. This model assumes that a given transposon family was present in many copies in the ancestor species. After species divergence, the transposon family is active at different levels in the two species (100 transpositions in one and 200 in the other species). As described in Additional file 1: Figure S1, A- and B-type gametes are passed on to offspring in a 1:1 ratio. In a cross-species comparison which identifies transposons (additional sequences) which are present in one but absent in the other species, insertions in one species and excisions in the other will be detected. If a transposon family had different levels of activity in the two species since their divergence, insertion/excision ratios will deviate from the 2:1 ratio.

## Abbreviations

TE: Transposable element
TIR: Terminal inverted repeat
MITE: Miniature inverted transposable element
TSD: Target site duplication
ORF: Open reading frame
DSB: Double-strand break
NHEJ: Non-homologous end joining
MMEJ: Microhomology-mediated end joining
SSA: Single strand annealing
SDSA: Synthesis-dependent strand annealing
InDel: Insertion or deletion
