# Supplementary material for: Genome-wide comparison of Asian and African rice reveals high recent activity of DNA transposons
Source: Mob DNA. 2015 Apr 28;6:8. doi: 10.1186/s13100-015-0040-x (PMC4423477; doi:10.1186/s13100-015-0040-x)
Supplement: Additional file 1: Figure S1. — Overview of the different mechanisms of DSB repair following DNA transposon excision. A.) Non-homologous end joining (NHEJ) which leads to perfect excisions. B.) Single stranded annealing (SSA) which leads to a deletion of adjacent sequences. C.) Synthesis-dependent strand annealing (SDSA) which can lead to introduction of ‘filler’ DNA segments. D.) A special case of SSA which leads to precise excisions. [file 13100_2015_40_MOESM1_ESM.pdf]

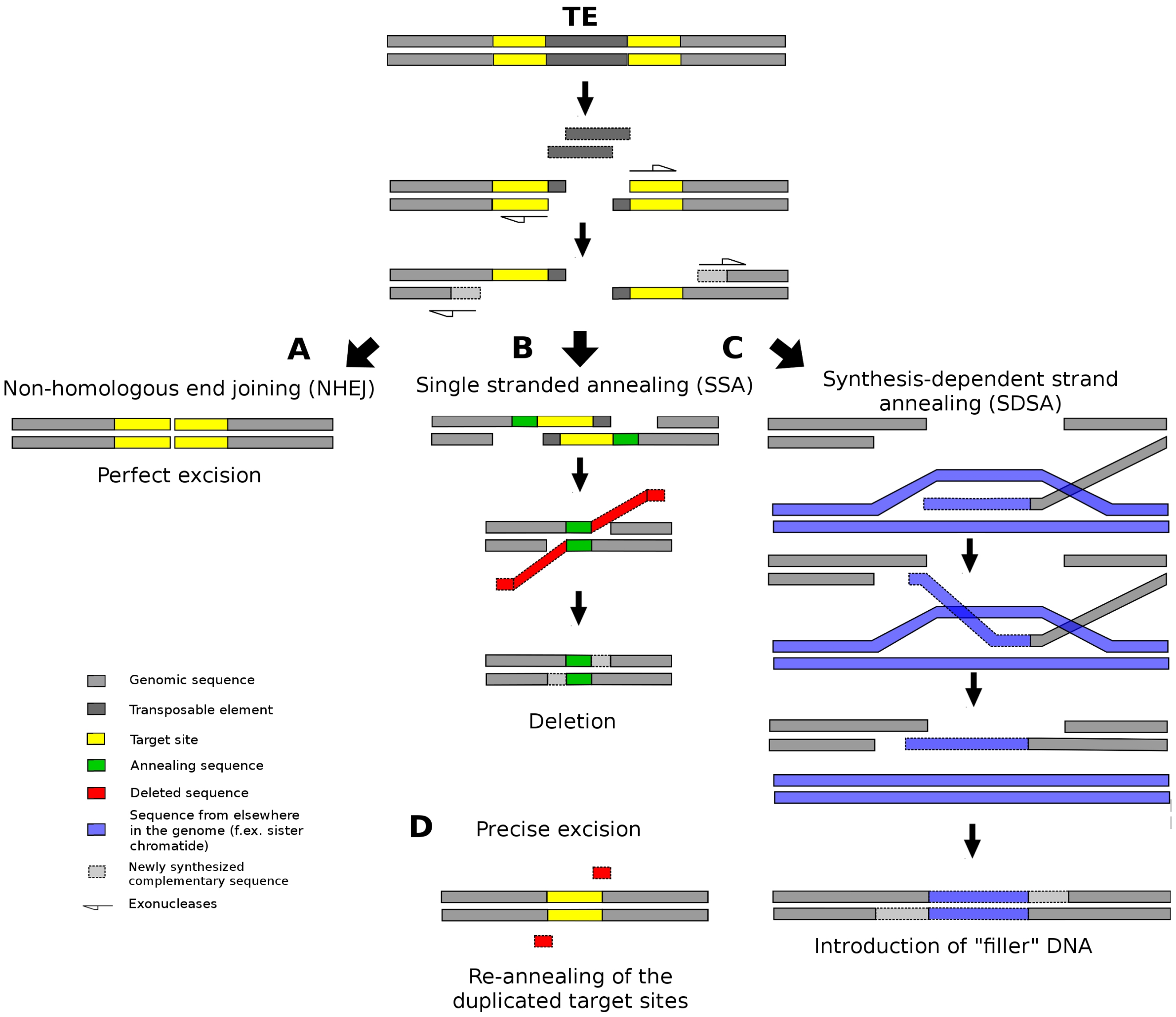

Additional Figure S1. Overview of the different mechanisms of DSB repair following DNA transposon excision. A.) Non-homologous end joining (NHEJ) which leads to perfect excisions. B.) Single stranded annealing (SSA) which leads to a deletion of adjacent sequences. C.) Synthesis-dependent strand annealing (SDSA) which can lead to introduction of “filler” DNA segments. D.) A special case of SSA which leads to precise

excisions.
