# Supplementary material for: Genome-wide comparison of Asian and African rice reveals high recent activity of DNA transposons
Source: Mob DNA. 2015 Apr 28;6:8. doi: 10.1186/s13100-015-0040-x (PMC4423477; doi:10.1186/s13100-015-0040-x)
Supplement: Additional file 2: Table S1. — Overview of TE abundance in O. sativa and O. glaberrima. [file 13100_2015_40_MOESM2_ESM.pdf]

Table S1: Overview of TE abundance in *O. sativa* and *O. glaberrima*

TE family	Copy number	Basepairs occupied	TE family	Copy number	Basepairs occupied
Mariner (DTT)			***Mariner (DTT)***		
DTT_SA	1875	220102	*DTT_SA*	1648	181016
DTT_SAA	214	30225	*DTT_SAA*	195	27307
DTT_SAB	79	5532	*DTT_SAB*	88	6046
DTT_SAC	115	10490	*DTT_SAC*	106	9467
DTT_SAD	1123	227077	*DTT_SAD*	1017	204483
DTT_SAF	699	87565	*DTT_SAF*	556	64707
DTT_SB	3995	487174	*DTT_SB*	3624	436358
DTT_SC	684	189223	*DTT_SC*	587	158365
DTT_SD	411	80172	*DTT_SD*	376	70226
DTT_SE	905	208112	*DTT_SE*	811	176028
DTT_SG	2403	461442	*DTT_SG*	2057	369602
DTT_SH	2060	347765	*DTT_SH*	1717	276194
DTT_SI	1063	213576	*DTT_SI*	834	155155
DTT_SJ	2354	247948	*DTT_SJ*	1809	184912
DTT_SK	496	101186	*DTT_SK*	423	84172
DTT_SM	2066	311754	*DTT_SM*	1842	272368
DTT_SN	241	53984	*DTT_SN*	223	47322
DTT_SQ	362	101019	*DTT_SQ*	332	91256
DTT_SR	25	1466	*DTT_SR*	22	1405
DTT_SS	803	151830	*DTT_SS*	674	126381
DTT_ST	612	57018	*DTT_ST*	528	50283
DTT_SU	148	31354	*DTT_SU*	134	26756
DTT_SV	173	14586	*DTT_SV*	140	11312
DTT_SW	978	177161	*DTT_SW*	892	160136
DTT_SX	304	50554	*DTT_SX*	285	44965
Harbinger (DTH)			***Harbinger (DTH)***		
DTH_Baba	52	14208	*DTH_Baba*	53	18994
DTH_Blip	68	51555	*DTH_Blip_A*	55	24292
DTH_OsKong	57	25927	*DTH_Kong*	62	32367
DTH_Pong	19	34467	*DTH_Pong*	9	861
DTH_TA	467	113832	*DTH_TA*	464	111840
DTH_TAA	288	68227	*DTH_TAA*	242	56944
DTH_TAB	143	26155	*DTH_TAB*	141	24325
DTH_TAC	48	6988	*DTH_TAC*	34	4959
DTH_TAD	1078	131160	*DTH_TAD*	877	104864
DTH_TAE	3022	332569	*DTH_TAE*	2426	254810
DTH_TAF	22	1733	*DTH_TAF*	17	1268
DTH_TAG	45	43781	*DTH_TAG*	25	15312
DTH_TAH	19	7408	*DTH_TAH*	24	34369
DTH_TAI	3028	601035	*DTH_TAI*	2764	539866
DTH_TAJ	179	29184	*DTH_TAJ*	192	32186
DTH_TAK	2	172	*DTH_TAK*	3	343
DTH_TAL	793	88893	*DTH_TAL*	672	73764
DTH_TAO	20	3591	*DTH_TAO*	19	3790
DTH_TAP	14	3840	*DTH_TAP*	11	2492
DTH_TAS	146	20824	*DTH_TAS*	129	18983
DTH_TAU	161	29966	*DTH_TAU*	159	29709
DTH_TB	109	13418	*DTH_TB*	100	13224

DTH_TC	1479	396387	*DTH_TC*	1202	301632
DTH_TD	131	9861	*DTH_TD*	131	9989
DTH_TE	510	123104	*DTH_TE*	457	110322
DTH_TF	1919	510105	*DTH_TF*	1745	452018
DTH_TG	937	165320	*DTH_TG*	796	135686
DTH_TI	93	8782	*DTH_TI*	89	8309
DTH_TO	3216	649618	*DTH_TO*	2955	595783
DTH_TR	581	148381	*DTH_TR*	399	99413
DTH_TS	1604	382053	*DTH_TS*	1183	264822
DTH_TT	505	43261	*DTH_TT*	389	33702
DTH_TU	103	11833	*DTH_TU*	93	11767
DTH_TV	224	34958	*DTH_TV*	207	32864
DTH_TW	50	9428	*DTH_TW*	83	18463
DTH_TY	152	26244	*DTH_TY*	102	16642
DTH_TZ	147	69196	*DTH_TZ*	133	56429
DTH_XAB	240	31875	*DTH_XAB*	228	30750
DTH_TX	294	92639	*DTH_TX*	261	76059
Mutator (DTM)			***Mutator (DTM)***		
DTM_HA	63	20859	*DTM_HA*	71	22988
DTM_MA	302	65813	*DTM_MA*	336	63054
DTM_MAA	212	60199	*DTM_MAA*	155	37006
DTM_MAB	86	11320	*DTM_MAB*	79	11603
DTM_MAC	308	56505	*DTM_MAC*	263	47824
DTM_MAD	2	1460	*DTM_MAD*	1	725
DTM_MAE	110	39384	*DTM_MAE*	79	27487
DTM_MAF	1408	234586	*DTM_MAF*	1232	207802
DTM_MAG	296	38061	*DTM_MAG*	265	32721
DTM_MB	202	94427	*DTM_MB*	196	93935
DTM_MC	251	138810	*DTM_MC*	255	89392
DTM_MD	152	47055	*DTM_MD*	143	42924
DTM_ME	622	110170	*DTM_ME*	542	93838
DTM_MF	64	27840	*DTM_MF*	78	32919
DTM_MG	74	29566	*DTM_MG*	46	26597
DTM_MH	78	11493	*DTM_MH*	74	10580
DTM_MJ	165	21874	*DTM_MJ*	124	15785
DTM_MK	672	1449063	*DTM_MK*	296	203120
DTM_MN	903	111517	*DTM_MN*	785	89989
DTM_MP	200	54956	*DTM_MP*	161	40220
DTM_MQ	118	48022	*DTM_MQ*	74	25182
DTM_MR	113	20488	*DTM_MR*	87	14383
DTM_MS	4	2385	*DTM_MS*	8	6766
DTM_MT	5	1286	*DTM_MT*	12	2522
DTM_MU	131	26646	*DTM_MU*	126	20587
DTM_MX	293	30976	*DTM_MX*	269	27413
DTM_MY	37	6092	*DTM_MY*	29	4729
DTM_MZ	123	13345	*DTM_MZ*	113	13898
DTM_XB	326	44185	*DTM_XB*	305	40352
CACTA (DTC)			***CACTA (DTC)***		
DTC_Alix	784	393380	*DTC_Alix*	533	176935
DTC_Baldur	21	30015	*DTC_Baldur*	29	20414
DTC_Benito	148	97417	*DTC_Benito*	140	81445
DTC_CA	188	44182	*DTC_CA*	155	39953
DTC_Calvin	4868	5511869	*DTC_Calvin*	3242	2099607
DTC_Carson	217	38003	*DTC_Carson*	193	30451

DTC_Dorian	369	462820	*DTC_Dorian*	275	185957
DTC_Eric	910	985533	*DTC_Eric*	818	764744
DTC_Grover	622	899717	*DTC_Grover*	406	358852
DTC_Isidor	285	39942	*DTC_Isidor*	262	23194
DTC_Janus	63	80190	*DTC_Janus*	55	47559
DTC_Radon	674	200825	*DTC_Radon*	313	93148
DTC_Rufus	22	43900	*DTC_Rufus*	22	14399
DTC_Sandro	67	36126	*DTC_Sandro*	91	77862
DTC_Seamus	20	28388	*DTC_Seamus*	14	10328
DTC_Sherman	137	60215	*DTC_Sherman*	125	47558
DTC_Storm	209	51912	*DTC_Storm*	198	53153
hAT (DTA)			***hAT (DTA)***		
DTA_Coraline	6	15292	*DTA_Coraline*	0	0
DTA_HA	16	5391	*DTA_HA*	7	3248
DTA_HB	192	25657	*DTA_HB*	172	24116
DTA_HC	122	19064	*DTA_HC*	108	16330
DTA_HD	482	112939	*DTA_HD*	379	71570
DTA_HE	244	22866	*DTA_HE*	219	20097
DTA_HG	32	4265	*DTA_HG*	37	7501
DTA_HI	22	3841	*DTA_HI*	12	1654
DTA_HJ	141	32099	*DTA_HJ*	118	26180
DTA_HK	92	45528	*DTA_HK*	59	16344
DTA_HL	73	8737	*DTA_HL*	49	5628
DTA_MI	146	20294	*DTA_MI*	162	23166
