# Supplementary material for: Genome-wide comparison of Asian and African rice reveals high recent activity of DNA transposons
Source: Mob DNA. 2015 Apr 28;6:8. doi: 10.1186/s13100-015-0040-x (PMC4423477; doi:10.1186/s13100-015-0040-x)
Supplement: Additional file 3: Figure S2. — Summary of causes for insertions in rice species. Many of the identified insertions showed homology with DNA transposons but were not caused directly by their activity (for example, partial deletions of TEs). Therefore, we divided the remaining insertions into three classes based on their presumed molecular mechanism as follows: (i) repeat slippage, (ii) partial deletion, and (iii) unknown. Repeat slippage happens if DNA polymerase loses its template while synthesizing the new strand during replication and then re-adopts at a similar template close by. We found 149 insertions in O. glaberrima and 51 in O. sativa which represent differences in the number of tandem repeats between the two species. Template lengths ranged from simple dinucleotides to more than 20 bp. In two cases, entire TEs served as templates for slippage, deleting several kb between two elements. In these cases, unequal homologous cross over (similar to the mechanism that produces solo LTRs of retrotransposons) could be an alternative interpretation. Another 68 insertions in O. glaberrima and 94 in O. sativa resulted from partial deletions of TEs. These were deletions of apparently random segments within or close to TEs. Finally, 35 insertions in O. glaberrima and 66 in O. sativa could not be clearly classified. These InDels are often larger than the average InDel. These include cases where it was not possible to deduce the original, ancient state because, for example, multiple TEs were nested in these positions. Also included here are cases where a TE was found in the middle if a large insertion. These could potentially represent excisions which went along with deletions of large segments of the flanking sequence. [file 13100_2015_40_MOESM3_ESM.pdf]

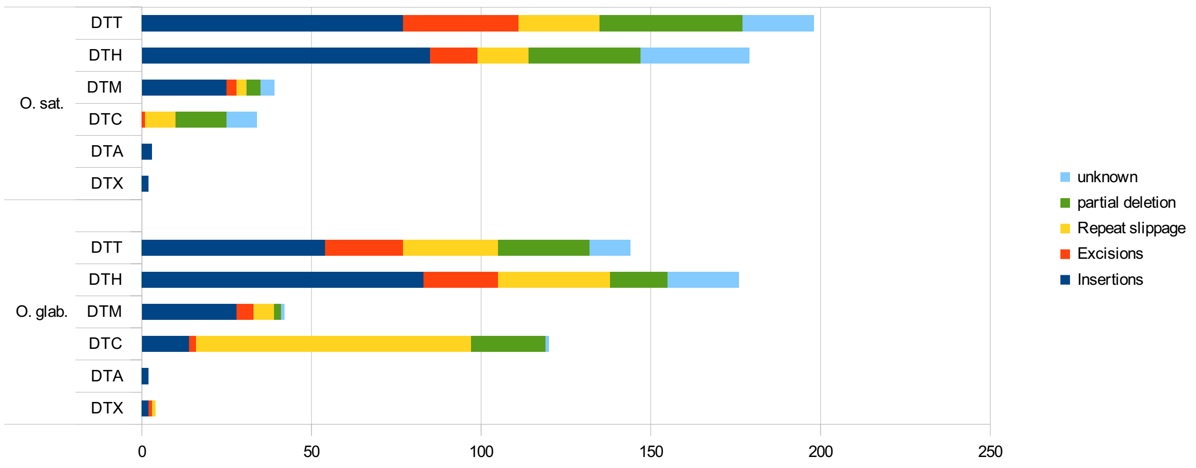

Additional Figure S2. Summary of causes for insertions in rice species. Many of the identified insertions showed homology with DNA transposons but were not caused directly by their activity (e.g. partial deletions of TEs). Therefore, were divided the remaining insertions in three classes based on their presumed molecular mechanism: (i) repeat slippage, (ii) partial deletion and (iii) unknown. Repeat slippage happens if DNA polymerase loses its template while synthesizing the new strand during replication and then re-adopts at a similar template close by. We found 149 insertion in *O. glaberrima* and 51 in the *O. sativa* which represent differences in the number of tandem repeats between the two species. Template lengths ranged from simple dinucleotides to more than 20 bp. In two cases, entire TEs served as templates for slippage, deleting several kb between two elements. In these cases, unequal homologous crossing-over (similar to the mechanism that produces solo LTRs of retrotransposons (ref)) could be an alternative interpretation. Another 68 insertions in *O. glaberrima* and 94 in *O. sativa*, resulted from partial deletions of TEs. These were deletions of apparently random segments within or close by TEs. Finally, 35 insertions in *O. glaberrima* and 66 in *O. sativa* could not be clearly classified. These indels are often larger than the average. These include cases where it was not possible to deduce the original, ancient state because, for example, multiple TEs were nested in these positions. Also included here are cases, where a TE was found in the middle if a large insertion. These could potentially represent excisions which went along with deletions of large segments of flanking sequence.
