# Supplementary material for: Genome-wide comparison of Asian and African rice reveals high recent activity of DNA transposons
Source: Mob DNA. 2015 Apr 28;6:8. doi: 10.1186/s13100-015-0040-x (PMC4423477; doi:10.1186/s13100-015-0040-x)
Supplement: Additional file 4: Table S2. — Overview of all transpositions. [file 13100_2015_40_MOESM4_ESM.pdf]

-

Table S2: Overview of all transpositions

ID	Excision / Insertion	TE family	Species	Chr.	Start *O. Sativa*	Start *O. glaberrima*	Target Site	bp deleted	bp „Filler“
1	E	*DTH_TS*	*O. sativa*	1	11361252	7664921	TAA	6	0
2	E	*DTM_MK*	*O. sativa*	1	12600385	9218754	CACCTCTTC / TCACCGTTCT	0	0
3	E	*DTH_TS*	*O. sativa*	1	12902570	9478733	TGA	0	13
4	E	*DTT_SAF*	*O. sativa*	1	14800923	10630380	TA	0	7
5	E	*DTT_SE*	*O. sativa*	1	2143974	1552674	TA	0	0
6	E	*DTT_SAF*	*O. sativa*	1	23428568	16010989	TA	17	16
7	E	*DTT_SI*	*O. sativa*	1	23745110	16234979	TA	5	0
8	E	*DTT_SH*	*O. sativa*	1	25116785	17535109	TA	0	1
9	E	*DTH_TR*	*O. sativa*	1	28103748	19840941	TAA	226	0
10	E	*DTT_SE*	*O. sativa*	1	28983077	20649311	TA	0	3
11	E	*DTT_SA*	*O. sativa*	1	29919153	21479533	TA	15	0
12	E	*DTM_MA*	*O. sativa*	1	33811302	24353085	ATGATAAAT	0	11
13	E	*DTH_TAS*	*O. sativa*	1	35000431	25182404	TTA	2	4
14	E	*DTC_Storm*	*O. sativa*	1	35288724	25434139	TAT	1	0
15	E	*DTH_TO*	*O. sativa*	1	41836087	31212136	TTA	10	2
16	E	*DTT_SA*	*O. sativa*	1	42564941	31717319	TA	0	1
17	E	*DTT_SG*	*O. sativa*	1	5150324	32601125	TA	5	0
18	E	*DTH_TF*	*O. sativa*	1	6649385	4959981	T(A/T)A	6	0
19	E	*DTT_SA*	*O. sativa*	2	11161618	9968245	TA	3	0
20	E	*DTT_SM*	*O. sativa*	2	1719100	1517049	TA	0	1
21	E	*DTT_SI*	*O. sativa*	2	21452665	17601179	TA	0	0
22	E	*DTH_TC*	*O. sativa*	2	21454670	17603056	TAA/TTC	5	0
23	E	*DTH_TA*	*O. sativa*	2	23428552	18987099	TT(T/A)	6	1
24	E	*DTT_SJ*	*O. sativa*	2	23974159	19453407	TA	3	2
25	E	*DTT_SG*	*O. sativa*	2	24072859	19545565	TA	23	161
26	E	*DTT_SH*	*O. sativa*	2	24169119	19638746	TA	4	3
27	E	*DTT_SJ*	*O. sativa*	2	27148252	21903384	TA	23	40
28	E	*DTT_SAF*	*O. sativa*	2	2725089	2463419	TA	0	2
29	E	*DTT_SC*	*O. sativa*	2	29194337	23380769	TA	0	12
30	E	*DTT_SG*	*O. sativa*	2	2963631	2706943	TA	14	0
31	E	*DTT_SJ*	*O. sativa*	2	29890827	23874746	TA	0	3
32	E	*DTT_SG*	*O. sativa*	2	33608046	27128831	TA	0	9
33	E	*DTH_TI*	*O. sativa*	2	34472772	27883970	T(G/A)G	12	2
34	E	*DTT_SJ*	*O. sativa*	2	35122138	28412790	TA	15	38
35	E	*DTH_TG*	*O. sativa*	2	400997	298568	TTA	3	0
36	E	*DTT_SM*	*O. sativa*	2	5051694	4634345	TA	0	1
37	E	*DTT_SJ*	*O. sativa*	2	5638072	5222271	TA	0	4
38	E	*DTT_SE*	*O. sativa*	2	6198541	5782264	TA	10	4
39	E	*DTT_SAF*	*O. sativa*	2	6453078	6059730	TA	121	6
40	E	*DTT_SI*	*O. sativa*	2	7026569	6419915	TA	0	1
41	E	*DTT_SC*	*O. sativa*	2	9717969	8789082	TA	0	1
42	E	*DTM_MH*	*O. sativa*	3	10435100	9646653	ATATATATATATGTAGAGAGA / TATATATAT	30	0
43	E	*DTT_SA*	*O. sativa*	3	11552786	10620135	TA	0	1
44	E	*DTT_SC*	*O. sativa*	3	12452418	11480541	TA	7	0
45	E	*DTH_TAA*	*O. sativa*	3	12884077	11897798	TAA	0	0
46	E	*DTT_SJ*	*O. sativa*	3	12918196	11942369	TA	16	4
47	I	*DTH_TG*	*O. sativa*	1	1029990	721289	TAA	0	0
48	I	*DTH_TAA*	*O. sativa*	1	10618137	8131027	TTA	0	0
49	I	*DTT_SJ*	*O. sativa*	1	1090465	786248	(T/C)A	0	0
50	I	*DTT_SJ*	*O. sativa*	1	1090465	786248	(T/C)A	0	0
51	I	*DTH_TC*	*O. sativa*	1	11079369	8420024	TAA	0	0
52	I	*DTH_TE*	*O. sativa*	1	11218178	7536702	TTA	0	0
53	I	*DTH_TAA*	*O. sativa*	1	11621340	32929750	TAA	0	0
54	I	*DTT_SG*	*O. sativa*	1	11675762	8504832	TA	0	0
55	I	*DTM_MK*	*O. sativa*	1	12105841	8797684	GAGCTGTCAA	0	0
56	I	*DTH_TS*	*O. sativa*	1	12350118	9044820	TTA	0	0
57	I	*DTT_SE*	*O. sativa*	1	12533001	9162528	TA	0	0
58	I	*DTM_MAA*	*O. sativa*	1	12664082	9283026	TTATTTTAA	0	0
59	I	*DTM_MAA*	*O. sativa*	1	12664082	9283026	TTATTTTAA	0	0
60	I	*DTH_TS*	*O. sativa*	1	12692397	9314114	CTC	0	0
61	I	*DTT_ST*	*O. sativa*	1	12714106	9326341	TA	0	0
62	I	*DTH_TY*	*O. sativa*	1	12864182	9437759	TTA	0	0
63	I	*DTT_SI*	*O. sativa*	1	13501578	9686190	TA	0	0
64	I	*DTM_MA*	*O. sativa*	1	14126284	10258650	GTCT(T/A)AACC	0	0
65	I	*DTH_TG*	*O. sativa*	1	14215209	10308991	TTA	0	0
66	I	*DTT_SAF*	*O. sativa*	1	14798576	10627374	TA	0	0
67	I	*DTC_Calvin*	*O. sativa*	1	15342434	10707383	TTA	0	0
68	I	*DTH_TS*	*O. sativa*	1	15345607	10709775	TAA	0	0
69	I	*DTC_Calvin*	*O. sativa*	1	15595678	10925172	TGG	0	0
70	I	*DTC_Alix*	*O. sativa*	1	15903567	11201038	GAA	0	0
71	I	*DTC_Benito*	*O. sativa*	1	17775468	12554804	CAC	0	0

-

72	I	*DTM_MK*	*O. sativa*	1	17931123	12708519	CGTGAATAGA	0	0
73	I	*DTH_TF*	*O. sativa*	1	18852422	13491709	TCA	0	0
74	I	*DTT_SAF*	*O. sativa*	1	18924946	13542799	TA	0	0
75	I	*DTT_SW*	*O. sativa*	1	19311706	13788640	TA	0	0
76	I	*DTT_SA*	*O. sativa*	1	19693153	14193910	TA	0	0
77	I	*DTH_Blip_A*	*O. sativa*	1	20703120	14567773	TAA	0	0
78	I	*DTH_TAI*	*O. sativa*	1	21066090	14732026	TAA	0	0
79	I	*DTT_SJ*	*O. sativa*	1	2139659	1548391	TA	0	0
80	I	*DTM_MA*	*O. sativa*	1	21500172	15074350	TACGGAGAT	0	0
81	I	*DTT_SA*	*O. sativa*	1	21567491	15121703	TA	0	0
82	I	*DTA_HJ*	*O. sativa*	1	21713076	15206684	AAATAATA	0	0
83	I	*DTH_TC*	*O. sativa*	1	21716777	15210337	TTA	0	0
84	I	*DTT_SJ*	*O. sativa*	1	21962983	15450955	TA	0	0
85	I	*DTH_TR*	*O. sativa*	1	22011604	15512019	TCA	0	0
86	I	*DTH_TE*	*O. sativa*	1	22023762	15526376	TTC	0	0
87	I	*DTT_SG*	*O. sativa*	1	22242282	15723374	TA	0	0
88	I	*DTH_TC*	*O. sativa*	1	23690198	16180807	TTA	0	0
89	I	*DTC_Calvin*	*O. sativa*	1	23709314	16195468	CTT	0	0
90	I	*DTT_SAF*	*O. sativa*	1	23993262	16435693	TA	0	0
91	I	*DTT_SG*	*O. sativa*	1	24012962	16451074	TA	0	0
92	I	*DTT_SJ*	*O. sativa*	1	24046421	16482148	TA	0	0
93	I	*DTC_Calvin*	*O. sativa*	1	24046828	16482349	AGG	0	0
94	I	*DTH_TC*	*O. sativa*	1	24139167	16578059	TAA	0	0
95	I	*DTH_TAO*	*O. sativa*	1	24409463	16831028	TTA	0	0
96	I	*DTH_TE*	*O. sativa*	1	24511445	16932491	TAA	0	0
97	I	*DTH_TR*	*O. sativa*	1	24777404	17209381	TTA	0	0
98	I	*DTH_TAA*	*O. sativa*	1	24832193	17264284	TTA	0	0
99	I	*DTC_Calvin*	*O. sativa*	1	25208673	17622358	ATT	0	0
100	I	*DTH_TS*	*O. sativa*	1	25478459	17886088	TTA	0	0
101	I	*DTH_OsKong*	*O. sativa*	1	25587961	17971265	TTA	0	0
102	I	*DTT_SH*	*O. sativa*	1	25929275	18125239	TA	0	0
103	I	*DTH_TG*	*O. sativa*	1	25996960	18182003	TAA	0	0
104	I	*DTC_Calvin*	*O. sativa*	1	26149708	18304811	TAA	0	0
105	I	*DTT_SA*	*O. sativa*	1	26194275	18340826	TA	0	0
106	I	*DTM_MC*	*O. sativa*	1	26232617	18365233	TTATAAAAT	0	0
107	I	*DTH_TC*	*O. sativa*	1	26615893	18676645	TAA	0	0
108	I	*DTH_TR*	*O. sativa*	1	26793849	18849018	TAA	0	0
109	I	*DTM_MAB*	*O. sativa*	1	26800778	18857349	CCCAAAATA	0	0
110	I	*DTT_SJ*	*O. sativa*	1	270582	195165	TA	0	0
111	I	*DTC_Calvin*	*O. sativa*	1	26969062	18978096	GGA	0	0
112	I	*DTH_TR*	*O. sativa*	1	27007522	18995540	TAA	0	0
113	I	*DTC_Benito*	*O. sativa*	1	27056979	19052371	GTA	0	0
114	I	*DTH_TC*	*O. sativa*	1	27302063	19242698	TTA	0	0
115	I	*DTH_TY*	*O. sativa*	1	27432878	19345246	TAA	0	0
116	I	*DTH_TAP*	*O. sativa*	1	2753187	2075913	TTA	0	0
117	I	*DTT_SAF*	*O. sativa*	1	27810679	19645995	TA	0	0
118	I	*DTC_Calvin*	*O. sativa*	1	27844500	19675740	GTT	0	0
119	I	*DTM_MA*	*O. sativa*	1	28092931	19833547	CTTTTATTT	0	0
120	I	*DTC_Grover*	*O. sativa*	1	28175915	19900871	AAG	0	0
121	I	*DTH_TC*	*O. sativa*	1	2805867	2129304	TAA	0	0
122	I	*DTH_TAD*	*O. sativa*	1	2806533	2129625	TGA	0	0
123	I	*DTH_TR*	*O. sativa*	1	28682974	20360981	TTA	0	0
124	I	*DTT_SK*	*O. sativa*	1	28699841	20376114	TA	0	0
125	I	*DTH_TR*	*O. sativa*	1	28764004	20443867	TAA	12	0
126	I	*DTH_TC*	*O. sativa*	1	28818278	20498889	TAA	0	0
127	I	*DTT_SG*	*O. sativa*	1	2871106	2194949	TA	0	0
128	I	*DTT_SAF*	*O. sativa*	1	28939358	20609588	TA	0	0
129	I	*DTT_SG*	*O. sativa*	1	28969568	20640436	TA	0	0
130	I	*DTH_TAI*	*O. sativa*	1	29000318	20666546	TTA	0	0
131	I	*DTM_MK*	*O. sativa*	1	29025892	20701769	CACTCTGTT	0	0
132	I	*DTH_TAA*	*O. sativa*	1	29144938	20797095	TTA	0	0
133	I	*DTH_TR*	*O. sativa*	1	29536779	21095477	TAA	0	0
134	I	*DTH_TAA*	*O. sativa*	1	29539166	21097583	TTA	0	0
135	I	*DTA_HI*	*O. sativa*	1	29663519	21227687	ATTGTATT	0	0
136	I	*DTH_TAA*	*O. sativa*	1	29777774	21309387	TTA	0	0
137	I	*DTT_SH*	*O. sativa*	1	29898371	21457014	TA	0	0
138	I	*DTH_TO*	*O. sativa*	1	29900074	21458466	TAA	0	0
139	I	*DTT_SI*	*O. sativa*	1	30637091	21978760	TA	0	0
140	I	*DTT_SJ*	*O. sativa*	1	3070643	2354740	TA	0	0
141	I	*DTH_TS*	*O. sativa*	1	30827768	22046978	TCA	0	0
142	I	*DTT_SH*	*O. sativa*	1	3137939	2412828	TA	0	0
143	I	*DTT_SG*	*O. sativa*	1	31363808	22341475	TA	0	0
144	I	*DTM_MK*	*O. sativa*	1	31513352	22474800	TTAGTATTAT / TTAGTACTA	1	0
145	I	*DTH_TAC*	*O. sativa*	1	32072664	22852509	TTA	0	0
146	I	*DTT_SC*	*O. sativa*	1	32526213	23235177	TA	0	0
147	I	*DTH_TR*	*O. sativa*	1	32527477	23236148	TTA	0	0
148	I	*DTT_SX*	*O. sativa*	1	32747903	23426262	TA	0	0
149	I	*DTM_MN*	*O. sativa*	1	32770865	23449069	GCTACAGAA	0	0
150	I	*DTT_SG*	*O. sativa*	1	32773764	23451346	TA	0	0
151	I	*DTM_MU*	*O. sativa*	1	32793489	23471138	GAATTTGAA	0	0

152	I	*DTH_TAA*	*O. sativa*	1	32807445	23480472	TAT	0	0
153	I	*DTH_TO*	*O. sativa*	1	32809218	23481180	TTA	0	0
154	I	*DTH_TC*	*O. sativa*	1	32822430	23481287	TAA	0	0
155	I	*DTH_TS*	*O. sativa*	1	32824934	23483357	TTA	0	0
156	I	*DTH_TR*	*O. sativa*	1	33177825	23827330	TAA	0	0
157	I	*DTH_TR*	*O. sativa*	1	33412795	24043583	TAA	0	0
158	I	*DTH_TR*	*O. sativa*	1	33413061	24043849	TAA	0	0
159	I	*DTM_MAD*	*O. sativa*	1	33414482	24044744	TTTAAATTT / TTTTTTTAATTT	3	0
160	I	*DTT_SH*	*O. sativa*	1	33507535	24098832	TA	0	0
161	I	*DTM_MK*	*O. sativa*	1	33531341	24123419	TAGACCCGA	0	0
162	I	*DTC_Calvin*	*O. sativa*	1	33667361	24212843	CGC	0	0
163	I	*DTT_SA*	*O. sativa*	1	34275147	24687769	TA	0	0
164	I	*DTA_HK*	*O. sativa*	1	34506671	24875789	CTCAGGGC(T/C)	0	0
165	I	*DTH_TO*	*O. sativa*	1	34801275	24980553	TTA	0	0
166	I	*DTT_SM*	*O. sativa*	1	34813658	24993091	TA	0	0
167	I	*DTH_TAF*	*O. sativa*	1	3489803	2723711	TTA	0	0
168	I	*DTM_MP*	*O. sativa*	1	34978639	25162648	CGCGGTGCA	0	0
169	I	*DTT_SA*	*O. sativa*	1	34999123	25181361	TA	0	0
170	I	*DTM_MQ*	*O. sativa*	1	35106477	25274490	ACTAGCAGA	0	0
171	I	*DTH_TR*	*O. sativa*	1	35109128	25276535	TA(A/G)	0	0
172	I	*DTT_SC*	*O. sativa*	1	35110147	25277210	TA	0	0
173	I	*DTH_TR*	*O. sativa*	1	35194415	25351719	TAA	0	0
174	I	*DTH_TS*	*O. sativa*	1	35515368	25640062	TAA	0	0
175	I	*DTC_Grover*	*O. sativa*	1	35792566	25870869	TTT	0	0
176	I	*DTM_MK*	*O. sativa*	1	35922945	24813066	ACGGTTAGC	0	0
177	I	*DTH_TC*	*O. sativa*	1	36042918	26185229	TTA	0	0
178	I	*DTT_SJ*	*O. sativa*	1	3622426	22511772	TA	0	0
179	I	*DTA_Coraline*	*O. sativa*	1	3622629	22511975	CGGAAACC	0	0
180	I	*DTM_MA*	*O. sativa*	1	36319685	26371502	ATAAATGAG	0	0
181	I	*DTH_TC*	*O. sativa*	1	3637091	22489351	TCA	0	0
182	I	*DTM_MAE*	*O. sativa*	1	36837071	26870950	GACTCTATG / TAGACTCTATG	2	0
183	I	*DTH_TR*	*O. sativa*	1	37301352	27211559	TTA	0	0
184	I	*DTH_TC*	*O. sativa*	1	37803012	27664111	TTC	0	0
185	I	*DTH_TX*	*O. sativa*	1	38202775	28020955	GTT	0	0
186	I	*DTH_TC*	*O. sativa*	1	38306346	28121068	TCA	0	0
187	I	*DTH_TC*	*O. sativa*	1	38384786	28198855	TTA	0	0
188	I	*DTH_TC*	*O. sativa*	1	38488039	28312458	T(C/T)A	0	0
189	I	*DTM_MR*	*O. sativa*	1	39183038	28963448	ACAATATAA	0	0
190	I	*DTM_MA*	*O. sativa*	1	39235137	29002088	AACTTGATG	0	0
191	I	*DTT_SG*	*O. sativa*	1	393663	302172	TA	0	0
192	I	*DTH_Kong*	*O. sativa*	1	39536580	29286687	TTA	0	0
193	I	*DTH_TR*	*O. sativa*	1	394030	302302	TT(A/C)	0	0
194	I	*DTH_TF*	*O. sativa*	1	39751967	29485011	TTA	0	0
195	I	*DTM_MA*	*O. sativa*	1	40450026	30134455	GTGTATTTA	0	0
196	I	*DTH_TG*	*O. sativa*	1	40743012	30404581	TAA	0	0
197	I	*DTT_SI*	*O. sativa*	1	41058142	30493453	TA	0	0
198	I	*DTM_MA*	*O. sativa*	1	41635434	31073955	TTTATGCAG	0	0
199	I	*DTT_SI*	*O. sativa*	1	41861760	31239649	TA	0	0
200	I	*DTM_MU*	*O. sativa*	1	41872930	31250773	CATAAGTAA	0	0
201	I	*DTH_TS*	*O. sativa*	1	4180877	3397035	TAA	0	0
202	I	*DTT_SG*	*O. sativa*	1	42167895	31436994	TA	0	0
203	I	*DTH_TE*	*O. sativa*	1	42217169	31482342	TTA	0	0
204	I	*DTT_SJ*	*O. sativa*	1	42228825	31493868	TA	0	0
205	I	*DTH_TX*	*O. sativa*	1	42265420	31530204	TTA	0	0
206	I	*DTH_TO*	*O. sativa*	1	42371967	31623913	TTA	0	0
207	I	*DTH_TA*	*O. sativa*	1	42564757	31717272	TAA	0	0
208	I	*DTT_SC*	*O. sativa*	1	4248654	3451587	TA	0	0
209	I	*DTM_MA*	*O. sativa*	1	42968458	32086827	TTAATTAGA	0	0
210	I	*DTT_SG*	*O. sativa*	1	43049231	32152731	TA	0	0
211	I	*DTT_SA*	*O. sativa*	1	43071509	32176237	TA	0	0
212	I	*DTM_MB*	*O. sativa*	1	43494073	32515318	TAAGTATT(G/A)	0	0
213	I	*DTH_TG*	*O. sativa*	1	4576059	3708481	TTA	0	0
214	I	*DTT_SX*	*O. sativa*	1	4828828	3865470	TA	0	0
215	I	*DTM_MK*	*O. sativa*	1	4836302	3873930	GTCCTATAT	0	0
216	I	*DTH_TR*	*O. sativa*	1	5084440	4092388	TAA	0	0
217	I	*DTT_SA*	*O. sativa*	1	5148338	32599016	TA	0	0
218	I	*DTH_TS*	*O. sativa*	1	5270639	21558334	TTA	0	0
219	I	*DTT_SI*	*O. sativa*	1	5274465	21561821	TA	0	0
220	I	*DTH_TS*	*O. sativa*	1	5362817	4316810	TTA	0	0
221	I	*DTM_MK*	*O. sativa*	1	543859	433660	TAATAGGAG	0	0
222	I	*DTT_SG*	*O. sativa*	1	5525433	4466706	TA	0	0
223	I	*DTH_TF*	*O. sativa*	1	577920	473181	CAA	0	0
224	I	*DTT_SG*	*O. sativa*	1	612468	506197	TA	0	0
225	I	*DTH_TS*	*O. sativa*	1	6217023	4568566	TAA	0	0
226	I	*DTT_SG*	*O. sativa*	1	6282708	4634701	TA	0	0
227	I	*DTH_TAA*	*O. sativa*	1	6437811	4763951	TTA	0	0
228	I	*DTM_MK*	*O. sativa*	1	723601	593019	GTGCAAACG	0	0
229	I	*DTT_SJ*	*O. sativa*	1	7270743	5514266	TA	0	0
230	I	*DTT_SG*	*O. sativa*	1	7303595	5542142	TA	0	0

231	I	*DTH_TR*	*O. sativa*	1	7499952	5693290	TTA	0	0
232	I	*DTT_SI*	*O. sativa*	1	78791	33903	TA	0	0
233	I	*DTT_SJ*	*O. sativa*	1	8022493	6149028	TA	0	0
234	I	*DTT_SC*	*O. sativa*	1	8084430	6224065	TA	0	0
235	I	*DTH_TO*	*O. sativa*	1	8795709	6889216	TAA	0	0
236	I	*DTA_HC*	*O. sativa*	1	8966844	7025200	ATCAGAAC	0	0
237	I	*DTT_SH*	*O. sativa*	1	9105981	7143780	TA	0	0
238	I	*DTH_TB*	*O. sativa*	1	9690186	7307353	TAA	0	0
239	E	*DTH_TA*	*O. glaberrima*	1	10528347	8032425	TAA	4	85
240	E	*DTH_TG*	*O. glaberrima*	1	10934179	8286779	GGT/ATA	0	0
241	E	*DTT_SD*	*O. glaberrima*	1	12632720	9251948	TA	10	0
242	E	*DTH_TO*	*O. glaberrima*	1	1339109	961268	TAA	0	40
243	E	*DTC_Calvin*	*O. glaberrima*	1	15197276	10707364	TTA	0	33
244	E	*DTM_MAC*	*O. glaberrima*	1	17556651	12361801	AAAATTAAA	1	0
245	E	*DTT_SA*	*O. glaberrima*	1	20924040	14704564	TA	1	1
246	E	*DTT_SQ*	*O. glaberrima*	1	23493205	16063204	TA	5	6
247	E	*DTH_TC*	*O. glaberrima*	1	25566676	17945502	TTA	0	100
248	E	*DTM_MAA*	*O. glaberrima*	1	25594551	17977187	TATAATTAA	18	11
249	E	*DTH_TG*	*O. glaberrima*	1	26810539	18866867	TAA	0	13
250	E	*DTH_TAE*	*O. glaberrima*	1	269507	194331	TTA	0	1
251	E	*DTM_MK*	*O. glaberrima*	1	26894172	18917971	TCAGAGTTC	0	15
252	E	*DTH_TO*	*O. glaberrima*	1	27695981	19606401	TTA	0	0
253	E	*DTH_TG*	*O. glaberrima*	1	27698927	19608776	TA(C/A)	0	9
254	E	*DTT_SE*	*O. glaberrima*	1	27706971	19614664	TA	0	0
255	E	*DTT_SJ*	*O. glaberrima*	1	27807094	19642683	TA	0	2
256	E	*DTT_SS*	*O. glaberrima*	1	28400216	20070370	TA	18	0
257	E	*DTT_SJ*	*O. glaberrima*	1	28465845	20139126	TA	0	1
258	E	*DTT_SA*	*O. glaberrima*	1	28760719	20441417	TA	6	0
259	E	*DTH_TO*	*O. glaberrima*	1	29925102	21491078	TTA	5	0
260	E	*DTT_SAF*	*O. glaberrima*	1	304763	211339	TA	0	10
261	E	*DTH_TR*	*O. glaberrima*	1	32417805	23123249	TTA	5	0
262	E	*DTH_TG*	*O. glaberrima*	1	32844031	23502369	TTA	22	0
263	E	*DTH_TS*	*O. glaberrima*	1	32995743	23645250	CTT/AAT	0	0
264	E	*DTT_SC*	*O. glaberrima*	1	34727057	24894673	TA	2479	2
265	E	*DTM_MK*	*O. glaberrima*	1	37710749	27573608	CTTGGGCGG / GTTTCTAA	0	19
266	E	*DTT_SG*	*O. glaberrima*	1	3855089	3064188	TA	3	4
267	E	*DTH_TG*	*O. glaberrima*	1	38726913	28525853	TAA	1	1
268	E	*DTT_SI*	*O. glaberrima*	1	39091831	28887455	TA	0	16
269	E	*DTH_TAG*	*O. glaberrima*	1	39194889	28973586	TTA	0	0
270	E	*DTT_SG*	*O. glaberrima*	1	397579	22916371	TA	7	0
271	E	*DTH_TR*	*O. glaberrima*	1	3995117	3217237	TTA	19	3
272	E	*DTH_TS*	*O. glaberrima*	1	40162275	29861404	TAA	1	0
273	E	*DTT_SI*	*O. glaberrima*	1	40377109	30069044	TA	3	2
274	E	*DTH_TG*	*O. glaberrima*	1	41420991	30853691	TAA	1	0
275	E	*DTT_SG*	*O. glaberrima*	1	41435175	30867749	TA	5	14
276	E	*DTH_TO*	*O. glaberrima*	1	41475508	30910997	TAA	3	0
277	E	*DTT_SM*	*O. glaberrima*	1	41552023	31001343	TA	0	1
278	E	*DTH_TR*	*O. glaberrima*	1	41647894	31084472	TAA	94	0
279	E	*DTM_MK*	*O. glaberrima*	1	42159590	31430923	TTTCCAACT	12	44
280	E	*DTT_SA*	*O. glaberrima*	1	42290224	31555204	TA	0	0
281	E	*DTT_SJ*	*O. glaberrima*	1	4445243	3618838	TA	0	5
282	E	*DTT_SI*	*O. glaberrima*	1	7088336	5381933	TA	0	1
283	E	*DTT_SG*	*O. glaberrima*	1	856012	627122	TA	7	9
284	E	*DTM_MAG*	*O. glaberrima*	1	8527114	6669527	TTACTAGTA	15	7
285	E	*DTM_MK*	*O. glaberrima*	1	8801090	6894249	CCATCTATA	14	2
286	E	*DTT_SJ*	*O. glaberrima*	2	35123671	28414477	TA	54	123
287	I	*DTH_TO*	*O. glaberrima*	1	10704193	8215296	TTA	0	0
288	I	*DTT_ST*	*O. glaberrima*	1	10875441	8234201	TA	0	0
289	I	*DTH_TW*	*O. glaberrima*	1	10980969	8327093	T(G/A)A	0	0
290	I	*DTM_XB*	*O. glaberrima*	1	11654383	8483867	(T/A)TATTAATT	0	0
291	I	*DTA_MI*	*O. glaberrima*	1	11677212	8506567	TTAG(A/C)ATT	0	0
292	I	*DTT_SV*	*O. glaberrima*	1	12635710	9254649	TA	0	0
293	I	*DTH_TF*	*O. glaberrima*	1	12680243	9299302	TAA	0	0
294	I	*DTM_MZ*	*O. glaberrima*	1	14801139	10630682	TTTTTAAAA	0	0
295	I	*DTM_MA*	*O. glaberrima*	1	16513446	11400157	ATGTTTCAA	0	0
296	I	*DTH_TO*	*O. glaberrima*	1	17703359	12488522	TCA	0	0
297	I	*DTT_SA*	*O. glaberrima*	1	18924818	13542482	TA	0	0
298	I	*DTH_TO*	*O. glaberrima*	1	20306244	14356850	TAA	0	0
299	I	*DTH_TG*	*O. glaberrima*	1	20578842	14468760	TTA	0	0
300	I	*DTT_SJ*	*O. glaberrima*	1	2143553	1552015	TA	0	0
301	I	*DTT_SA*	*O. glaberrima*	1	21677622	15174678	TA	0	0
302	I	*DTT_SAF*	*O. glaberrima*	1	21968452	15456544	TA	0	0
303	I	*DTT_SG*	*O. glaberrima*	1	22039788	15542210	TA	0	0
304	I	*DTM_MD*	*O. glaberrima*	1	22043909	15547010	TTTTAAAAA	0	0
305	I	*DTH_TO*	*O. glaberrima*	1	23434952	16017266	TAA	0	0
306	I	*DTT_SG*	*O. glaberrima*	1	23744590	16234223	TA	0	0
307	I	*DTH_TC*	*O. glaberrima*	1	24002093	16440426	TTA	0	0
308	I	*DTH_TG*	*O. glaberrima*	1	24019574	16458436	TAA	0	0
309	I	*DTM_MC*	*O. glaberrima*	1	24091648	16527920	ATTCTTCTT	0	0

310	I	*DTT_ST*	*O. glaberrima*	1	24171930	16611975	TA	0	0
311	I	*DTM_MA*	*O. glaberrima*	1	24692360	17122961	TTATCAGTA	0	0
312	I	*DTH_TC*	*O. glaberrima*	1	25100147	17523297	TAA	0	0
313	I	*DTT_SJ*	*O. glaberrima*	1	25194761	17608772	TA	0	0
314	I	*DTM_MA*	*O. glaberrima*	1	25347445	17764949	AAGAAGCAG	0	0
315	I	*DTM_MA*	*O. glaberrima*	1	25600673	17983050	GTAGTTAAC	0	0
316	I	*DTM_MAF*	*O. glaberrima*	1	25945747	18135717	AGCTTTCAT	0	0
317	I	*DTA_HL*	*O. glaberrima*	1	25945579	18135549	CTTG(C/T)GTC	0	0
318	I	*DTM_MA*	*O. glaberrima*	1	2609560	1947187	TTATAGTAG	0	0
319	I	*DTH_TAA*	*O. glaberrima*	1	26160512	18314521	TTA	0	0
320	I	*DTH_TS*	*O. glaberrima*	1	26386988	18496175	TAA	0	0
321	I	*DTH_TW*	*O. glaberrima*	1	27575049	19486786	TAA	0	0
322	I	*DTT_SC*	*O. glaberrima*	1	28157347	19879800	TA	0	0
323	I	*DTH_TAA*	*O. glaberrima*	1	28395887	20065798	TTA	0	0
324	I	*DTH_TO*	*O. glaberrima*	1	28481996	20155402	TTA	0	0
325	I	*DTT_SJ*	*O. glaberrima*	1	28705765	20384749	TA	0	0
326	I	*DTH_TF*	*O. glaberrima*	1	28708553	20388052	AAG	0	0
327	I	*DTT_SAF*	*O. glaberrima*	1	2874183	2197794	TA	0	0
328	I	*DTT_SE*	*O. glaberrima*	1	29263895	20875104	TA	0	0
329	I	*DTT_SV*	*O. glaberrima*	1	29314897	20927422	(G/T)A	3	1
330	I	*DTM_MA*	*O. glaberrima*	1	29432840	20999747	CAGAATCAA	0	0
331	I	*DTT_SW*	*O. glaberrima*	1	29912091	21473105	TA	0	0
332	I	*DTH_TS*	*O. glaberrima*	1	30003221	21627040	T(A/G)A	0	0
333	I	*DTH_OsKong*	*O. glaberrima*	1	309517	215967	ATA	0	0
334	I	*DTH_TO*	*O. glaberrima*	1	31313547	22303314	TTA	0	0
335	I	*DTT_SAF*	*O. glaberrima*	1	3139572	2414996	TA	0	0
336	I	*DTH_TG*	*O. glaberrima*	1	3178114	2430641	TAG	0	0
337	I	*DTH_TO*	*O. glaberrima*	1	32063578	22843354	TAA	0	0
338	I	*DTH_TO*	*O. glaberrima*	1	3259560	2482799	TTA	0	0
339	I	*DTH_TAA*	*O. glaberrima*	1	33276263	28018512	TAA	0	0
340	I	*DTT_SA*	*O. glaberrima*	1	3339618	2568157	TA	0	0
341	I	*DTT_SAF*	*O. glaberrima*	1	33583786	24151489	TA	0	0
342	I	*DTM_MA*	*O. glaberrima*	1	34065115	24508580	CTGCCTGGCA	0	0
343	I	*DTH_TO*	*O. glaberrima*	1	34822235	25001296	TTA	0	0
344	I	*DTH_TR*	*O. glaberrima*	1	3484655	2718328	TAA	0	0
345	I	*DTH_TO*	*O. glaberrima*	1	3550993	2785464	TAA	0	0
346	I	*DTH_TAJ*	*O. glaberrima*	1	35550255	25669499	TTA	0	0
347	I	*DTH_TAA*	*O. glaberrima*	1	35647397	25747014	ATA	0	0
348	I	*DTT_SC*	*O. glaberrima*	1	3594651	2831363	TA	0	0
349	I	*DTH_TAK*	*O. glaberrima*	1	3706080	22299004	AAT	0	0
350	I	*DTT_SJ*	*O. glaberrima*	1	37296379	27207210	TA	0	0
351	I	*DTH_TR*	*O. glaberrima*	1	3732134	2969271	TTA	0	0
352	I	*DTT_SJ*	*O. glaberrima*	1	3812501	3018181	TA	0	0
353	I	*DTT_SAF*	*O. glaberrima*	1	3814401	3020306	TA	0	0
354	I	*DTH_TC*	*O. glaberrima*	1	3836196	3042879	TTA	0	0
355	I	*DTA_MI*	*O. glaberrima*	1	38506328	28327375	ACTGGGGC	0	0
356	I	*DTH_TO*	*O. glaberrima*	1	38530142	28352552	TAA	0	0
357	I	*DTH_TAL*	*O. glaberrima*	1	3853727	3062262	(C/T)AA	0	0
358	I	*DTT_SJ*	*O. glaberrima*	1	39049480	28844172	TA	0	0
359	I	*DTM_MG*	*O. glaberrima*	1	39500304	29246445	TTGGGTCCC	0	0
360	I	*DTH_TAA*	*O. glaberrima*	1	39810394	29545484	TTA	0	0
361	I	*DTA_HG*	*O. glaberrima*	1	40648068	30313114	GTGCCAAC	0	0
362	I	*DTT_SG*	*O. glaberrima*	1	40744936	30406282	TA	0	0
363	I	*DTT_SD*	*O. glaberrima*	1	4109883	3324756	TA	0	0
364	I	*DTM_MA*	*O. glaberrima*	1	41353649	30791667	GTATATGTA	0	0
365	I	*DTH_TO*	*O. glaberrima*	1	41474060	30909102	TAA	0	0
366	I	*DTT_SAF*	*O. glaberrima*	1	41481931	30917534	TA	0	0
367	I	*DTT_SJ*	*O. glaberrima*	1	42035990	31336175	TA	0	0
368	I	*DTH_TC*	*O. glaberrima*	1	42140161	31410262	TTA	0	0
369	I	*DTH_TAA*	*O. glaberrima*	1	42273321	31537912	TTA	0	0
370	I	*DTH_TW*	*O. glaberrima*	1	42381359	31633231	TTT	0	0
371	I	*DTT_SH*	*O. glaberrima*	1	42694478	31840334	TA	0	0
372	I	*DTT_SW*	*O. glaberrima*	1	42696092	31843109	TA	0	0
373	I	*DTH_TF*	*O. glaberrima*	1	42696204	31843221	TCA	0	0
374	I	*DTH_TG*	*O. glaberrima*	1	43069280	32174013	AGA	0	0
375	I	*DTH_TC*	*O. glaberrima*	1	43468568	32488744	TAA	0	0
376	I	*DTH_TX*	*O. glaberrima*	1	445049	347768	TTA	0	0
377	I	*DTT_SG*	*O. glaberrima*	1	454212	23075768	TA	0	0
378	I	*DTT_SH*	*O. glaberrima*	1	5138352	4145127	TA	0	0
379	I	*DTH_TE*	*O. glaberrima*	1	6436479	4762202	TTA	0	0
380	I	*DTM_MA*	*O. glaberrima*	1	6513527	4837806	CTTATCCAG	0	0
381	I	*DTT_SG*	*O. glaberrima*	1	655715	562531	TA	0	0
382	I	*DTH_TW*	*O. glaberrima*	1	6672110	4982838	TGA	0	0
383	I	*DTT_SH*	*O. glaberrima*	1	6712193	5023409	TA	0	0
384	I	*DTM_MA*	*O. glaberrima*	1	6993028	5274232	ATCATCAGG	0	0
385	I	*DTH_TAI*	*O. glaberrima*	1	7099266	5392844	TAA	0	0
386	I	*DTT_SAF*	*O. glaberrima*	1	7688404	5846504	TA	0	0
387	I	*DTT_SAF*	*O. glaberrima*	1	8199790	6312623	TA	0	0
388	I	*DTH_TO*	*O. glaberrima*	1	8211756	6325553	TTA	0	0
389	I	*DTT_SH*	*O. glaberrima*	1	8315235	6420875	TA	0	0

390	I	*DTT_SC*	*O. glaberrima*	1	8405774	6513637	(C/T)A	0	0
391	I	*DTH_TC*	*O. glaberrima*	1	9105923	7143130	GAA	0	0
392	I	*DTT_SS*	*O. glaberrima*	2	10553904	9437687	TA	0	0
393	I	*DTT_SN*	*O. glaberrima*	2	10896325	9736395	TA	0	0
394	I	*DTM_MA*	*O. glaberrima*	2	1097217	938744	AATGCATAA	0	0
395	I	*DTT_SC*	*O. glaberrima*	2	11169256	9976064	TA	0	0
396	I	*DTT_SAF*	*O. glaberrima*	2	1394755	1210004	TA	0	0
397	I	*DTT_SJ*	*O. glaberrima*	2	1441626	1258434	TA	0	0
398	I	*DTH_TAA*	*O. glaberrima*	2	14666538	12263634	TTA	0	0
399	I	*DTT_SC*	*O. glaberrima*	2	15674241	13440855	TA	0	0
400	I	*DTT_SI*	*O. glaberrima*	2	17168914	14466443	TA	0	0
401	I	*DTT_SG*	*O. glaberrima*	2	17192207	14497769	TA	0	0
402	I	*DTT_SAC*	*O. glaberrima*	2	17196700	14503820	TA	0	0
403	I	*DTH_TC*	*O. glaberrima*	2	17225372	14531805	TAA	0	0
404	I	*DTH_TAH*	*O. glaberrima*	2	17330937	14599964	AAT	0	0
405	I	*DTH_TX*	*O. glaberrima*	2	18602093	15133289	TTA	0	0
406	I	*DTH_TG*	*O. glaberrima*	2	18714532	15238083	TTA	0	0
407	I	*DTT_SC*	*O. glaberrima*	2	18743498	15260630	TA	0	0
408	I	*DTH_TAA*	*O. glaberrima*	2	19230476	15622651	T(C/T)A	0	0
409	I	*DTM_MA*	*O. glaberrima*	2	19443668	15840060	TCATAGGAC	0	0
410	I	*DTH_OsKong*	*O. glaberrima*	2	19593695	15944034	(C/T)AA	0	0
411	I	*DTT_SJ*	*O. glaberrima*	2	1970138	1827656	TA	0	0
412	I	*DTH_TO*	*O. glaberrima*	2	19833459	16144180	TTA	0	0
413	I	*DTT_SAF*	*O. glaberrima*	2	19840237	16154011	T(G/A)	0	0
414	I	*DTM_MS*	*O. glaberrima*	2	19966452	16281051	TTTCCTGGG	0	0
415	I	*DTH_TS*	*O. glaberrima*	2	19973007	16291414	TAA	0	0
416	I	*DTH_TR*	*O. glaberrima*	2	20255974	16590745	TTA	0	0
417	I	*DTT_SC*	*O. glaberrima*	2	20890086	17100541	TA	0	0
418	I	*DTH_TW*	*O. glaberrima*	2	22071350	18147549	TAA	0	0
419	I	*DTT_SAF*	*O. glaberrima*	2	22550672	20584672	TA	0	0
420	I	*DTH_TF*	*O. glaberrima*	2	23109331	18782894	TAA	0	0
421	I	*DTH_TA*	*O. glaberrima*	2	23448711	18987739	TAA	0	0
422	I	*DTT_SH*	*O. glaberrima*	2	23463034	19000706	TA	0	0
423	I	*DTT_SA*	*O. glaberrima*	2	23483987	19014058	TA	0	0
424	I	*DTT_SC*	*O. glaberrima*	2	23483987	19014058	TA	0	0
425	I	*DTH_XAB*	*O. glaberrima*	2	23483914	19013985	TTA	0	0
426	I	*DTT_SA*	*O. glaberrima*	2	23875953	19378042	TA	0	0
427	I	*DTT_SJ*	*O. glaberrima*	2	24223170	19693947	TA	0	0
428	I	*DTT_SA*	*O. glaberrima*	2	24556077	19946822	TA	0	0
429	I	*DTT_SC*	*O. glaberrima*	2	24788509	20153594	TA	0	0
430	I	*DTT_SA*	*O. glaberrima*	2	26269326	21160525	TA	0	0
431	I	*DTH_TS*	*O. glaberrima*	2	26298533	21190083	TAA	0	0
432	I	*DTH_TR*	*O. glaberrima*	2	2790606	2537937	TTA	0	0
433	I	*DTH_TC*	*O. glaberrima*	2	28324273	22784202	TCA	0	0
434	I	*DTH_TW*	*O. glaberrima*	2	29853205	23836422	TAA	0	0
435	I	*DTT_SA*	*O. glaberrima*	2	30381782	24334798	TA	0	0
436	I	*DTT_SG*	*O. glaberrima*	2	30595276	24547218	TA	0	0
437	I	*DTT_SQ*	*O. glaberrima*	2	30598443	24548787	TA	0	0
438	I	*DTM_MA*	*O. glaberrima*	2	30852649	24772080	TACTTAAAT	0	0
439	I	*DTH_TC*	*O. glaberrima*	2	30931533	24850090	TAA	0	0
440	I	*DTT_SX*	*O. glaberrima*	2	3316804	2998021	TA	0	0
441	I	*DTH_TO*	*O. glaberrima*	2	33403290	26932183	T(T/A)A	0	0
442	I	*DTT_SX*	*O. glaberrima*	2	34012993	27513144	TA	0	0
443	I	*DTH_TC*	*O. glaberrima*	2	34069760	27566980	TCA	0	0
444	I	*DTH_TG*	*O. glaberrima*	2	34390903	27813086	TT(G/A)	0	0
445	I	*DTH_TS*	*O. glaberrima*	2	34795955	28118299	ATA	0	0
446	I	*DTH_TAA*	*O. glaberrima*	2	35140828	28433640	TAA	0	0
447	I	*DTT_SC*	*O. glaberrima*	2	35499378	28755574	TA	0	0
448	I	*DTH_TC*	*O. glaberrima*	2	35775691	28976707	TCA	0	0
449	I	*DTH_TAA*	*O. glaberrima*	2	3757655	3442028	GAG	0	0
450	I	*DTT_SG*	*O. glaberrima*	2	408889	305338	TA	0	0
451	I	*DTH_TO*	*O. glaberrima*	2	4363382	3914524	TAA	0	0
452	I	*DTH_TO*	*O. glaberrima*	2	4749310	4315470	TAA	0	0
453	I	*DTH_TC*	*O. glaberrima*	2	5310357	4886128	GAA	0	0
454	I	*DTH_TF*	*O. glaberrima*	2	5380801	4954732	ATT	0	0
455	I	*DTH_TAU*	*O. glaberrima*	2	5657125	5242755	TAA	0	0
456	I	*DTT_SD*	*O. glaberrima*	2	5855634	5442020	TA	0	0
457	I	*DTH_TO*	*O. glaberrima*	2	5942793	5538952	TTA	0	0
458	I	*DTT_SI*	*O. glaberrima*	2	5955249	5569352	TA	0	0
459	I	*DTT_SH*	*O. glaberrima*	2	6252462	5839464	TA	0	0
460	I	*DTT_SA*	*O. glaberrima*	2	6314603	5917863	TA	0	0
461	I	*DTM_MA*	*O. glaberrima*	2	6762742	6141194	TACATATGG	0	0
462	I	*DTM_MD*	*O. glaberrima*	2	6783930	6161308	TTAAGGAAA	0	0
463	I	*DTT_SH*	*O. glaberrima*	2	7015546	6403859	TA	0	0
464	I	*DTT_SG*	*O. glaberrima*	2	7026769	6420238	TA	0	0
465	I	*DTH_TW*	*O. glaberrima*	2	7071607	6467288	T(T/A)A	0	0
466	I	*DTT_SC*	*O. glaberrima*	2	7305178	6709042	TA	0	0
467	I	*DTH_TC*	*O. glaberrima*	2	7760792	7165361	TTA	0	0
468	I	*DTM_MT*	*O. glaberrima*	2	8021904	7474342	TACCATTATGTA	0	0
469	I	*DTH_TB*	*O. glaberrima*	2	8299451	7733908	TTA	0	0

470	I	*DTT_SA*	*O. glaberrima*	2	8317260	7752000	TA	0	0
471	I	*DTM_MA*	*O. glaberrima*	2	9070596	8405297	TATTTATAA	0	0
472	I	*DTT_SE*	*O. glaberrima*	2	9118930	8435181	TA	0	0
473	I	*DTT_SH*	*O. glaberrima*	2	9411322	8553775	TA	0	0
474	I	*DTT_SA*	*O. glaberrima*	2	943835	801480	TA	0	0
475	I	*DTH_TAA*	*O. glaberrima*	3	10434766	9645591	(C/T)GA	0	0
476	I	*DTH_TW*	*O. glaberrima*	3	10925359	10103886	TCA	0	0
477	I	*DTH_TG*	*O. glaberrima*	3	10959877	10138641	ATA	0	0
478	I	*DTH_TS*	*O. glaberrima*	3	11460996	10512412	TTA	0	0
479	I	*DTH_TO*	*O. glaberrima*	3	11563899	10631536	TTA	0	0
480	I	*DTT_SI*	*O. glaberrima*	3	12372363	11394352	TA	0	0
481	I	*DTT_SG*	*O. glaberrima*	3	12372711	11394939	TA	0	0
482	I	*DTH_TAD*	*O. glaberrima*	3	12397058	11419392	TAG	0	0
483	I	*DTM_MAC*	*O. glaberrima*	3	12399247	11421625	TTTTTTTAA	0	0
484	I	*DTT_SG*	*O. glaberrima*	3	12435261	11463175	TA	0	0
485	I	*DTH_TAB*	*O. glaberrima*	3	12915389	11939220	TTA	0	0
486	I	*DTH_TW*	*O. glaberrima*	3	12931333	11956232	TAC	0	0
487	I	*DTM_HA*	*O. glaberrima*	3	12940204	11967304	CACCGAGAC	9	0
