# Supplementary material for: Genome-wide comparison of Asian and African rice reveals high recent activity of DNA transposons
Source: Mob DNA. 2015 Apr 28;6:8. doi: 10.1186/s13100-015-0040-x (PMC4423477; doi:10.1186/s13100-015-0040-x)
Supplement: Additional file 5: Figure S3. — Inheritance of transposon insertion/excision patterns. For this model we assume that all transposition effects are selectively neutral. It is commonly accepted that a mechanism of multiplication is for DNA transposons to excise during DNA replication and to reinsert in front of the replication fork. This leads to one daughter strand with one copy of the element (A-type gamete) and one with two copies (B-type gamete). If a large number of transposons are active in many different loci in a species (this may be spread out over many generations), the offspring genome will be a mosaic of loci derived from A- and B-type gametes. When comparing that genome to that of a closely related species, loci resulting from A-type gametes will identify an excision and an insertion, while loci resulting from A-type gametes will only identify insertions. Thus the observed overall ratio of insertions to excisions from a given transposon family will be 2:1. [file 13100_2015_40_MOESM5_ESM.pdf]

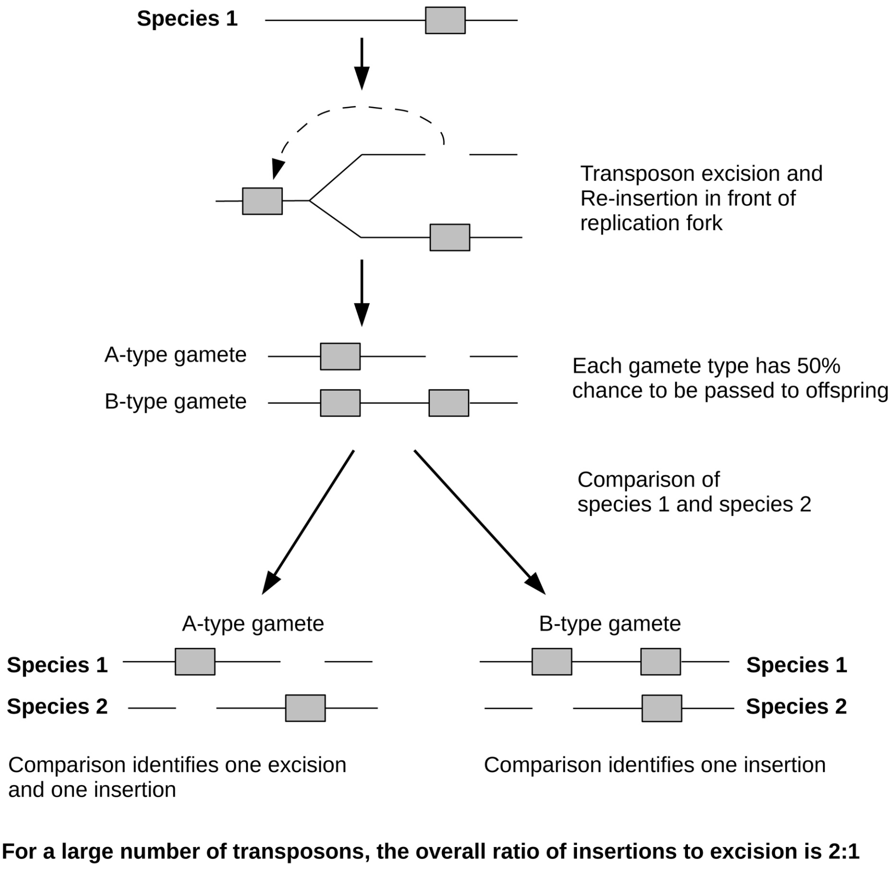

Supplementary Figure S3. Inheritance of transposon insertion/excision patterns. For this model we assume that all transposition effects are selectively neutral. It is commonly accepted that on mechanism of multiplication is for DNA transposons to excise during DNA replication and to re-insert in front of the replication fork. This leads to one daughter strand with one copy of the element (A-type gamete) and one with two copies (B-type gamete). If a large number of transposons are active in many different loci in a species (this may be spread out over many generations), the offspring genome will be a mosaic of loci derived from A and B-type gametes. When comparing that genome to that of a closely related species, loci resulting from A-type gametes will identify an excision and an insertion, while loci resulting from A-type gametes will only identify insertions. Thus the observed overall ratio of insertions to excisions fro a given transposon family will be 2:1.
