# Supplementary material for: Genome-wide comparison of Asian and African rice reveals high recent activity of DNA transposons
Source: Mob DNA. 2015 Apr 28;6:8. doi: 10.1186/s13100-015-0040-x (PMC4423477; doi:10.1186/s13100-015-0040-x)
Supplement: Additional file 6: Figure S4. — Detection of differences in transposon activity in different species. This model assumes that a given transposon family was present in many copies in the ancestor species. After species divergence, the transposon family is active at different levels in the two species (100 transpositions in one and 200 in the other species). As described in Additional file 1: Figure S1, A- and B-type gametes are passed on to offspring in a 1:1 ratio. In a cross-species comparison which identifies transposons (additional sequences) which are present in one but absent in the other species, insertions in one species and excisions in the other will be detected. If a transposon family had different levels of activity in the two species since their divergence, insertion/excision ratios will deviate from the 2:1 ratio. [file 13100_2015_40_MOESM6_ESM.pdf]

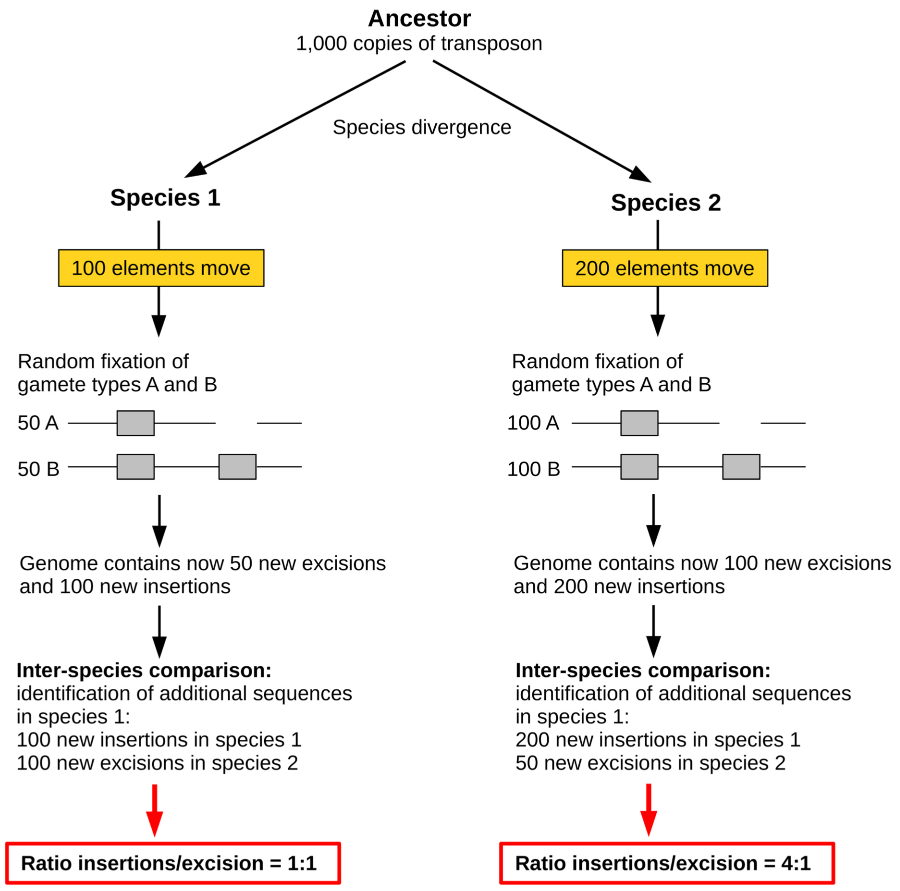

Supplementary Figure S4. Detection of differences in transposon activity in different species. This model assumes that a given transposon family was present in many copies in the ancestor species. After species divergence, the transposon family is active at different levels in the two species (100 transpositions in one and 200 in the other species). As described in Supplementary Figure 1, A and B-type gametes are passed on to offspring in a 1:1 ratio. In a cross-species comparison which identifies transposons (additional sequences) which are present in one but absent in the other species, insertions in one species and excisions in the other will be detected. If a transposon family had different levels of activity in the two species since their divergence, insertion/excision ratios will deviate from the 2:1 ratio.
